# Effects of 5-ion 6-beam sequential irradiation in the presence and absence of hindlimb or control hindlimb unloading on behavioral performances and plasma metabolic pathways of Fischer 344 rats

**DOI:** 10.3389/fphys.2024.1486767

**Published:** 2024-11-13

**Authors:** Jacob Raber, Mitali Chaudhari, Alexis De la Torre, Sarah Holden, Kat Kessler, Breanna Glaeser, Marek Lenarczyk, Scott Willem Leonard, Alexander Borg, Andy Kwok, Chirayu Patel, Amy Kronenberg, Christopher M. Olsen, Jeffrey S. Willey, Jeffrey Morré, Jaewoo Choi, Jan Frederik Stevens, Gerd Bobe, Jessica Minnier, John Baker

**Affiliations:** ^1^ Department of Behavioral Neuroscience, Oregon Health & Science University, Portland, OR, United States; ^2^ Departments of Neurology, Psychiatry, and Radiation Medicine, Division of Neuroscience ONPRC, Oregon Health and Science University, Portland, OR, United States; ^3^ College of Pharmacy, Oregon State University, Corvallis, OR, United States; ^4^ Neuroscience Center and Department of Pharmacology and Toxicology, Medical College of Wisconsin, Milwaukee, WI, United States; ^5^ Radiation Biosciences laboratory, Medical College of Wisconsin, Milwaukee, WI, United States; ^6^ Department of Radiation Oncology, Wake Forest School of Medicine, Winston-Salem, NC, United States; ^7^ Biological Systems and Engineering Division, Lawrence Berkeley National Laboratory, Berkeley, CA, United States; ^8^ Mass Spectrometry Center, Oregon State University, Corvallis, OR, United States; ^9^ Linus Pauling Institute, Oregon State University, Corvallis, OR, United States; ^10^ Department of Animal & Rangeland Sciences, Oregon State University, Corvallis, OR, United States

**Keywords:** Fischer rats, 5-ion beam irradiation, hindlimb unloading, open-field, object recognition, metabolomics

## Abstract

**Introduction:**

Effects and interactions between different spaceflight stressors are expected to be experienced by crew on missions when exposed to microgravity and galactic cosmic rays (GCRs). One of the limitations of previous studies on simulated weightlessness using hindlimb unloading (HU) is that a control HU condition was not included.

**Methods:**

We characterized the behavioral performance of male Fischer rats 2 months after sham or total body irradiation with a simplified 5-ion 6-mixed-beam exposure representative of GCRs in the absence or presence of HU. Six months later, the plasma, hippocampus, and cortex were processed to determine whether the behavioral effects were associated with long-term alterations in the metabolic pathways.

**Results:**

In the open field without and with objects, interactions were observed for radiation × HU. In the plasma of animals that were not under the HU or control HU condition, the riboflavin metabolic pathway was affected most for sham irradiation vs. 0.75 Gy exposure. Analysis of the effects of control HU on plasma in the sham-irradiated animals showed that the alanine, aspartate, glutamate, riboflavin, and glutamine metabolisms as well as arginine biosynthesis were affected. The effects of control HU on the hippocampus in the sham-irradiated animals showed that the phenylalanine, tyrosine, and tryptophan pathway was affected the most. Analysis of effects of 0.75 Gy irradiation on the cortex of control HU animals showed that the glutamine and glutamate metabolic pathway was affected similar to the hippocampus, while the riboflavin pathway was affected in animals that were not under the control HU condition. The effects of control HU on the cortex in sham-irradiated animals showed that the riboflavin metabolic pathway was affected. Animals receiving 0.75 Gy of irradiation showed impaired glutamine and glutamate metabolic pathway, whereas animals receiving 1.5 Gy of irradiation showed impaired riboflavin metabolic pathways. A total of 21 plasma metabolites were correlated with the behavioral measures, indicating that plasma and brain biomarkers associated with behavioral performance are dependent on the environmental conditions experienced.

**Discussion:**

Phenylalanine, tyrosine, and tryptophan metabolism as well as phenylalanine and tryptophan as plasma metabolites are biomarkers that can be considered for spaceflight as they were revealed in both Fischer and WAG/Rij rats exposed to simGCRsim and/or HU.

## 1 Introduction

Astronauts are frequently exposed to different types of ionizing radiations in space that could impact their brain functions (Braby et al., 2019). Microgravity is another environmental stressor experienced by astronauts during space missions that could cause brain dysfunction. Hindlimb unloading (HU) has been used to simulate microgravity on Earth (Ray et al., 2001) and shown to reduce the hippocampal levels of pyruvate dehydrogenase, which is a part of glucose metabolism and is associated with oxidative stress and brain ischemia, as well as the structural protein tubulin (Sarkar et al., 2006). In addition, microgravity achieved using parabolic flights has been shown to impair visuospatial tuning and orientation in mice during the flights (Oman, 2007). Irradiation and microgravity may also interact in the manner in which they cause DNA damage (Moreno-Villanueva et al., 2017) and other injury-related cellular pathways, including oxidative stress, mitochondrial functions (Yatagai et al., 2019), cardiovascular health (Patel, 2020), and bone functions (Krause et al., 2017), as well as the brain (Raber et al., 2021). Recently, we reported the complex interactions of simulated microgravity achieved by HU and a simplified field of simulated space radiation (SimGCRsim) on the behavioral and cognitive performances as well as metabolic pathways in the plasma and brain of WAG/Rij rats (Raber et al., 2021). Sham-irradiated WAG/Rij rats exposed to simulated microgravity with HU were impaired in terms of hippocampus-dependent spatial habituation learning, unlike irradiated WAG/Rij rats (1.5 Gy). In addition, rats exposed to 1.5 Gy of SimGCRsim showed increased depressive-like behaviors in the absence but not in the presence of simulated microgravity. Specific behavioral measures, such as activity and anxiety measures as well as spatial habituation in the open field and depressive-like behaviors in the forced swim test, were associated with the plasma levels of distinct metabolites 10 months after the behavioral testing. The phenylalanine, tyrosine, and tryptophan metabolic pathway was the one most profoundly affected by radiation in the absence and presence of microgravity in terms of the plasma and by microgravity itself.

One of the limitations of simulated spatialradiation studies in the absence or presence of simulated weightlessness using hindlimb unloading (HU) is the absence of a control HU condition. Therefore, to determine whether these effects of irradiation and simulated microgravity are specific to WAG/Rij rats and whether these effects are specific to simulated space radiation, we characterized the behavioral and cognitive performance of male Fischer rats 2 months after exposure to sham irradiation or total body irradiation with simGCRsim (0.75 or 1.5 Gy) in the absence or presence of simulated microgravity along with HU or control HU condition. In addition, we collected plasma samples 9 months after sham irradiation or total body irradiation and analyzed them for distinct alterations in the metabolic pathways to determine whether changes to the metabolic measures were associated with specific behavioral and cognitive measures. Finally, we compared the metabolic measures associated with specific behavioral and cognitive performances following simGCRsim exposure in the present study with the findings following photon irradiation in our previous study (Raber et al., 2024).

## 2 Materials and methods

### 2.1 Animals and radiation exposure

Male 7–8 months old Fischer 344 rats (RRID:RGD_734478) (*n* = 132, see Table 1 for details of the experimental groups) were shipped to the Brookhaven National Laboratory (BNL), Upton, NY, United States. Male rats were used in this study as they are an established model of radiation injury to the cardiovascular system (Baker et al., 2009), and the current study was based on extant studies. After 2 weeks of acclimatization and at 9 months of age, they were sham-irradiated or irradiated with a standardized simplified 5-ion 6-beam prescribed by NASA referred to as simGCRsim, which consists of sequential and rapidly switched exposures to protons (1000 MeV/n), ^28^Si ions (250 MeV/n), ^4^He ions (350 MeV/n), ^16^O ions (600 MeV/n), ^56^Fe ions (250 MeV/n), and protons (250 MeV/n) (0, 0.75, or 1.5 Gy) in the absence or presence of simulated weightlessness achieved by HU or a control HU condition. The control HU condition was identical to the HU condition with the exception that the animals could use all four paws for movement.

**TABLE 1:** Experimental radiation and HU groups.

Radiation dose (Gy)	HU	Number of rats for behavioral testing	Number of rats for metabolomics analysis
0	Sham	22	12
0.75	Sham	22	12
1.5	Sham	22	12
0	Control HU	12	12
0.75	Control HU	12	12
1.5	Control HU	12	12
0	HU	10	
0.75	HU	10	
1.5	HU	10	

The HU procedure was performed 5 days prior to sham-irradiation or irradiation, as described below. The rats remained in the HU condition for 25 days following sham-irradiation or irradiation. At the end of one week following the HU period, the animals were shipped to the Medical College of Wisconsin (MCW). The animals were then maintained on a Teklad 8904 diet (Indianapolis, IN, United States) and fed *ad libitum* during the study. The animals were housed in a reverse light cycle room with lights off from 07.30–19.30. All behavioral and cognitive tests were performed during the dark period starting 2 months after irradiation or sham-irradiation. All animal procedures were consistent with ARRIVE guidelines and were reviewed and approved by the Institutional Animal Care and Use Committees at BNL and MCW. All analyses were performed blinded to the treatment, and the code was revealed only after the data were analyzed.

### 2.2 HU

**FIGURE 1:**
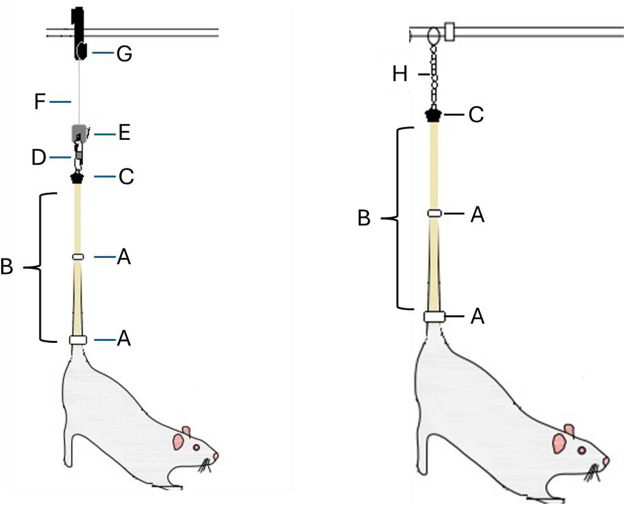
Schematic detailing the components associated with the tail suspension procedure for placement in the specialized hindlimb unloading (HU) housing cages (Left) or transfer to the NASA Space Radiation Laboratory (NSRL) for placement in the beamline (Right). Common components for both methods: [A] strips of wrap-around micropore tape; [B] medical traction tape applied along the lateral portion of the tail that is then wrapped through a hole in the bottom portion of [C], which is a specialized and custom-made swivel for HU housing cage-specific components (Left). [D] is a carabiner connected to the underlying swivel, and [E] is a zipper hook above, which is attached to and permits adjustment of the height of the rat through vertical movements along a [F] Perlon cord attached to a [G] pulley to permit sliding of the setup over a steel bar. Beamline housing-specific components (Right). [H] is a bead chain connected to the underlying swivel that is then connected to the grooves in the aluminum frame placed in the beamline.

On day 1, the rats were randomly grouped into full weightbearing (non-HU), HU via tail suspension, or no HU (sham). The HU procedure was a modified version of that published previously (Raber et al., 2021; Wiley et al., 2016) (Figure 1). In brief, the rats were anesthetized with isoflurane (2.5%–3.0% at 5% oxygen flow rate). Then, a tincture of benzoin was applied to the lateral surface of the tail before placing one of the free ends of an adhesive medical traction tape (3M, St. Paul, MN, United States) starting approximately 1 cm from the base of the tail and adhering up to 3/4th of the tail length. The free end was then looped through a catch on a plastic ball-bearing swivel and adhered to the opposite lateral side of the tail. Strips of micropore tape (3M) were adhered perpendicularly over the traction tape. A 1.56-inch carabiner clip (Nite Ize, Boulder, CO, United States) was connected to the swivel and a zipper hook to permit vertical adjustment along an attached Perlon cord (STAS group, BA, Eindhoven, the Netherlands). The cord was then connected to a pulley (Blue Hawk, Gilbert, AZ, United States) that permitted the setup to slide securely over a 5/16-inch-diameter steel rod (Hillman, Cincinnati, OH, United States). Adjustment of the zipper lock lifted the hind limbs off the substrate, with the abdomen and thorax suspended at 30° from the horizontal plane. The sham HU rats received isoflurane but were not tail suspended. For placement along the beamline (Figure 1) for both the irradiation and sham procedures, the swivel was disconnected from the carabiner clip, and a plastic bead chain cord (Luanxu, Guangzhou City, China) was looped through the swivel at the point of the carabiner attachment. The rats were then transferred to specialized rat boxes in 60 cm × 60 cm aluminum frames, and the bead chain lengths were adjusted to ensure that the hind limbs remained suspended at the same angle as in the HU cages. The bead chain was then placed in a slot at the top of the frame to ensure suspension of the rodent hind limbs, and the swivel permitted rotation in the boxes.

### 2.3 Performances in the open field in the absence and presence of objects (week 1)

Exploratory behaviors, measures of anxiety, and hippocampus-dependent spatial habituation learning were assessed in a black open field (90.49 cm × 90.49 cm × 45.72 cm) during two consecutive days, similar to that in the previous study with middle-aged male WAG/Rij rats (Raber et al., 2021). The animal cages were brought into the testing room; then, the rats were picked up gently by placing the thumb and forefinger behind the forelegs and wrapping the palm around the stomach. The researchers supported the animals by their stomachs during transportation to and from the enclosure. The animals were placed in the middle of the open field for 5 min each day. The researchers vacated the testing room during behavioral testing. Following the testing of each rat, the enclosure was cleaned with a solution of 70% isopropyl alcohol. The outcome measures analyzed using video tracking (AnyMaze software, Stoelting Co., Wood Dale, IL, United States) were the total distance moved, time spent freezing, and percentage of time spent in the more anxiety-provoking center zone (45.25 cm × 45.25 cm).

On day 3, the rats were tested in the open field containing 3D-printed objects based on the protocol, as reported previously (Aguilar et al., 2018), with the following modifications. The objects were placed 45.72 cm from the top as well as 30.48 cm from the left and right sides of the enclosure, with the distance between the two objects also being 30.48 cm. The rats were then placed in the enclosure containing two identical blue objects (squares) or two identical red objects (cylinders) for an acquisition trial of 5 min. The objects were counterbalanced for this task, where half the rats started with the blue squares and the other half started with the red cylinders. Sixty minutes following the acquisition trial (A), the rats were again placed in the enclosure with one of the familiar objects replaced with a novel red or blue object for the test trial (T). The outcome measures analyzed using video tracking were the total distance moved and percentage of time spent in the center zone (45.25 cm × 45.25 cm) containing the objects. In addition, the percentage of time that each animal explored the novel and familiar objects in the test trial was analyzed by manually scoring the digital videos. The light intensity during the open field and object recognition tests was 50 lux. A white noise generator (setting II) and overhead lights were used during testing.

### 2.4 Plasma metabolomics analysis

Nine months following sham-irradiation or irradiation and 7 months after the behavioral and cognitive tests, blood samples of 72 rats (Table 1) were collected in EDTA-containing tubes, the samples were centrifuged at 2,000*g* for 10 min, and the plasma supernatant was collected and stored at −80°C for plasma metabolomics. To compare the results of the current study with those of our earlier HU rat studies and due to COVID-19-related travel restrictions, the time points for the plasma analyses were not the same as those of the behavioral tests. The rats were euthanized by guillotine without anesthesia; the brain was then cut along the midline on ice in phosphate-buffered saline (PBS), and the entire hippocampus and complete cortex were dissected as described by Raber et al. (2002) before being stored at −80°C. The tissues were homogenized in 300 μL (per 30 μg of tissue) of cold methanol and water (8:2, v/v), and the metabolites were extracted from 100 μL of plasma. Untargeted metabolomic analysis was then performed as described by Kirkwood et al. (2013). Liquid chromatography (LC) was performed using a Shimadzu Nexera system with an insertil phenyl-3 column (4.6 × 150 mm, 100 Å, 5 μm; GL Sciences, Rolling Hills Estates, CA, United States) coupled to a quadrupole time-of-flight mass spectrometer (Q-TOF MS; AB SCIEX, Triple TOF 5600) operated in the information-dependent MS/MS acquisition mode. The samples were ordered randomly and included multiple quality control (QC) samples. To ensure stability and repeatability of the LC-MS system, we included QC samples comprising equal mixtures of 10 μL aliquots of the representative biological samples from the same study; after every 10 samples, a total of seven QC assessments were performed so that the analytical variations would be much smaller than the biological variability. The coefficient of variation was generally within 15% when the signal-to-noise ratio was >10. All samples were tested in both positive and negative ion modes. In the case that metabolites were present in both ion modes, the mode with the higher peak value was selected for further analysis. The column temperature was maintained at 50°C, and the samples were preserved at 10°C. To detect the metabolic features and normalize the data, the metabolomics data were processed using Markerview (version 1.3.1, SCIEX, Framingham, MA, United States) software. After removing the isotopologs and adducts of the same molecular species, the metabolite identities were assigned using Peakview software (SCIEX) by matching for accurate mass (error <10 ppm), retention time (error <10%), MS/MS fragmentation (library score >70), and isotope distribution (error <20%) using an in-house library consisting of IROA standards (IROA Technologies, Bolton, MA, United States) and other commercially available standards (650 total) for integrated pathway and statistical analyses. In addition to the IROA database, the fragmentation spectra of all peaks were verified with data from Metlin (Scripps, La Jolla, CA, United States) and HMDB (University of Alberta, Edmonton, Canada). This confirmation of the metabolites using retention time, mass-to-charge (m/z) ratio, and comparisons with authentic standards (±1 min) through the in-house library (IROA Technologies) allowed streamlined identification of the metabolites. LipidMaps (Welcome Trust, United Kingdom), Metlin, and HMDB were used for the MS and MS/MS matching. MetaboAnalyst pathway analysis (Montreal, Quebec, Canada) was performed as described earlier (Kirkwood et al., 2013; Xia and Wishart, 2010). The raw metabolomite peak values from the plasma, hippocampus, and cortex were analyzed by Pareto scaling through six distinct comparisons: 1) effects of radiation (0.75 Gy) compared to sham irradiation in rats without HU; 2) effects of radiation (1.5 Gy) compared to sham irradiation in rats without HU; 3) effects of radiation compared to sham irradiation, in rats with control HU; 4) effects of control HU in sham-irradiated rats: sham vs. control HU; 5) effects of control HU in 0.75-Gy-irradiated rats: sham vs. control HU; 6) effects of control HU in 1.5-Gy-irradiated rats: sham vs. control HU. The pathways were visualized using scatter plots (testing for significant features) in MetaboAnalyst with “global test” and “relative-betweenness centrality” as the parameters for the enrichment method and topological analysis, respectively.

### 2.5 Statistical analyses

All data were presented as means ± standard errors of the means (SEMs). The behavioral and cognitive data were analyzed with SPSS v.25 software (IBM, Armonk, NY, United States). To analyze the behavioral and cognitive performances after sham irradiation or simGCRsim irradiation (0, 0.75, or 1.5 Gy), we performed analyses of variance (ANOVAs) with *post hoc* tests comparing the sham-irradiated animals where appropriate. To assess the role of microgravity, we performed ANOVAs including the radiation (0, 0.75, or 1.5 Gy) and HU conditions (sham, control HU, or HU) as between-group factors with repeated measures where appropriate. For some analyses, as indicated and appropriate, two-sided *t*-tests were used. We set the statistical significance to *p* < 0.05. Greenhouse–Geisser corrections were used if the sphericity was found to be violated (Mauchly’s test).

The plasma metabolomics were analyzed for the comparisons described above. To identify the potential plasma biomarkers of radiation exposure or HU condition on behavioral or cognitive performance, we used regression analyses. Based on Pareto scaling, we selected the metabolites that were consistently included in the models. MetaboAnalyst software was used to generate the impact plots, and the graphs were generated using GraphPad software v.8.2.0 (La Jolla, CA, United States). As MetaboAnalyst is not ideal for analyzing amino-acid-related pathways, we analyzed them separately as described under the metabolomics analysis above.

## 3 Results

### 3.1 Effects of HU and radiation on performance in the open field

**FIGURE 2:**
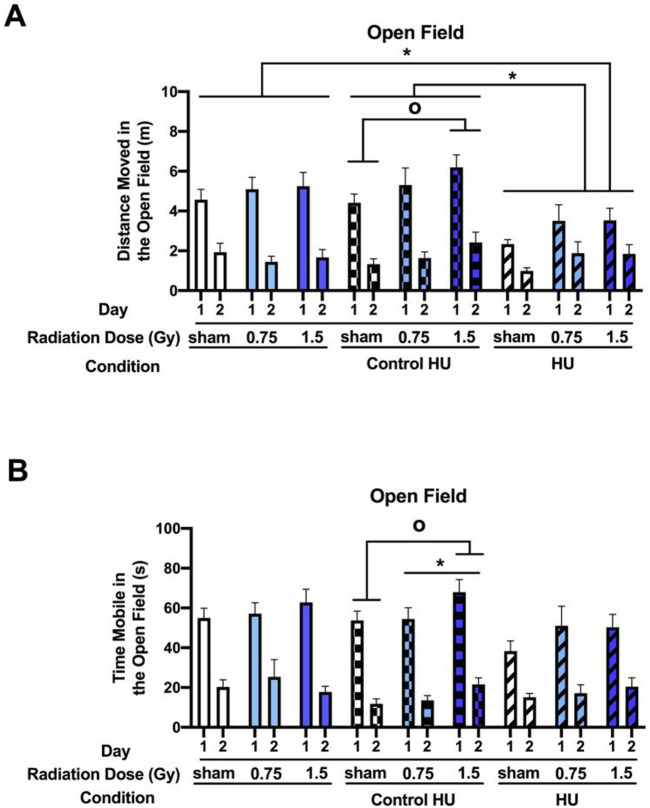
Performance in the open field in the absence of objects. The rats were tested for exploratory behaviors in the open field on two consecutive days, with the trials lasting 5 min each and spaced 24 h apart. **(A)** Analysis of the activity levels on two consecutive days in the open field showed a condition × day interaction (*F* (2, 123) = 6.367, *p* = 0.002). In the control HU group, activity levels were higher in animals exposed to 1.5 Gy than the sham-irradiated animals (^o^ *p* < 0.05, Dunnett). There was also a main effect of HU (*F* (2, 123) = 4.606, *p* = 0.012), with the animals under HU moving less than those under control HU or without HU (*p < 0.05, Tukey). **(B)** Analysis of the time spent mobile showed a similar pattern. In the control HU group, there was a radiation effect, with higher activity levels in animals exposed to 1.5 Gy than the sham-irradiated animals (*p < 0.05, Tukey; ^o^ *p* < 0.05, Dunnett). These effects were not observed in animals under HU or without HU.

When the activity levels were analyzed for two consecutive days in the open field, there was a condition × day interaction (*F* (day, condition) (2, 123) = 6.367, *p* = 0.002). In the control HU group, there was a trend toward radiation effects (*F* (2, 33) = 3.102, *p* = 0.058), with higher activity levels in animals irradiated with 1.5 Gy than the sham-irradiated animals (*p* = 0.0183, Dunnett’s test). There was also a main effect of HU on the activity level in the open field (*F* (2, 123) = 4.606, *p* = 0.012), with HU animals moving less than animals under control HU (*p* = 0.015, Tukey’s test) or no HU (*p* = 0.029, Tukey’s test). There was also a day effect (*F* (1, 123) = 150.240, *p* < 0.001), with lower activity levels on day 2 than day 1, indicating that all groups showed spatial habitation learning (Figure 2A). There was also a trend toward an overall effect of radiation that was not significant (*F* (2, 123) = 2.698, *p* = 0.071). 

Similar results were obtained for the analysis of the time spent mobile (Figure 2B). In the control HU group, there was a radiation effect (*F* (2, 33) = 3.775, *p* = 0.033), with higher activity levels in animals irradiated with 1.5 Gy than the sham-irradiated animals (*p* = 0.0161, Dunnett’s test); these effects were not observed in animals under the HU or no HU condition.

### 3.2 Effects of HU and radiation on performance in the open field with objects

**FIGURE 3:**
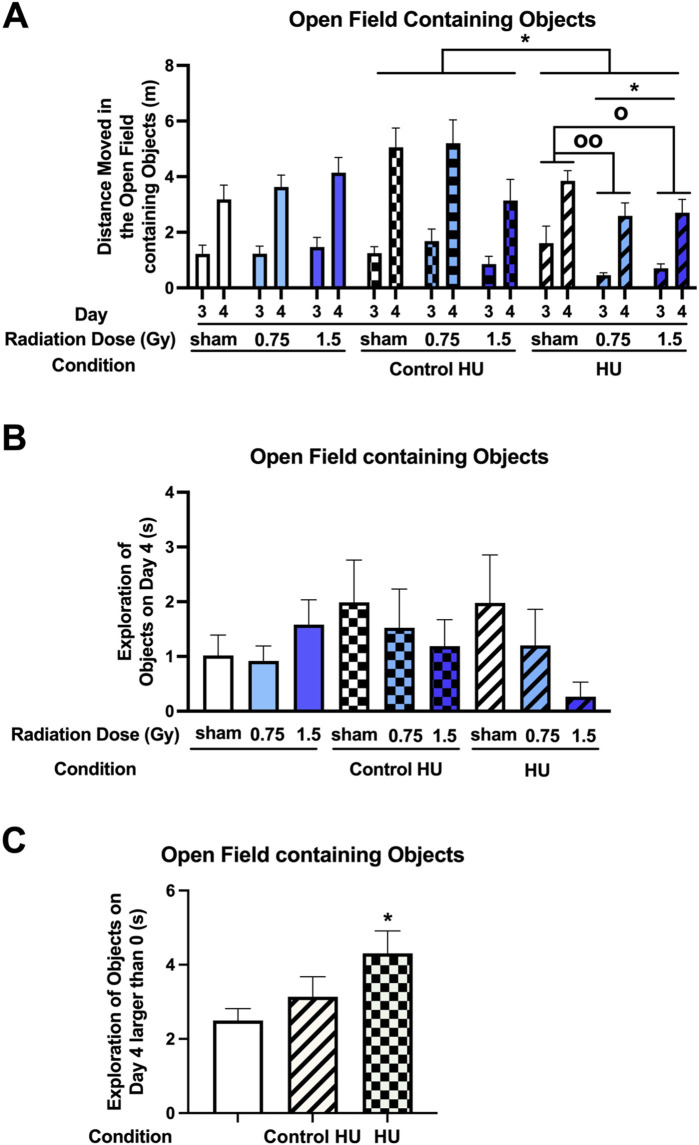
**(A)** Performance assessment in the open field containing objects showed that there was an effect of HU condition (*F* (2, 123) = 3.164, *p* = 0.046) and a radiation × HU condition interaction (*F* (4, 123) = 3.118, *p* = 0.018). Animals under HU moved less than animals under control HU (*p < 0.05, Tukey). In the HU group, there was a radiation effect (*F* (2, 27) = 4.704, *p* = 0.018); HU animals irradiated with 0.75 or 1.5 Gy moved less than the sham-irradiated HU animals (^o^ *p* < 0.05, ^oo^ *p* < 0.01, Dunnett). There was also a day effect (*F* (1, 123) = 136.269, *p* < 0.001), with higher activity levels on day 4 involving one familiar and one novel object than on day 3 involving two identical objects. **(B)** There was no effect of radiation or HU condition on the time spent exploring the objects on day 4. **(C)** When the rats that did not explore the objects on a particular day were excluded from the analysis, the animals under HU were noted to have spent more time exploring the objects than those without HU (*p < 0.05, Dunnett).

The activity levels were analyzed for two consecutive days in the open field containing objects. There was an effect of the HU condition (*F* (2, 123) = 3.164, *p* = 0.046) and a radiation × HU condition interaction (*F* (4, 123) = 3.118, *p* = 0.018) (Figure 3A). Animals under HU moved less than those under control HU (*p* = 0.035, Tukey’s test). In the HU group, there was a radiation effect (*F* (2, 27) = 4.704, *p* = 0.018); HU animals that were irradiated with 0.75 Gy (*p* = 0.0087, Dunnett’s test) or 1.5 Gy (*p* = 0.0283, Dunnett’s test) moved less than the sham-irradiated HU animals. In contrast, there were no significant effects of radiation in the control HU group (*F* (2, 33) = 2.950, *p* = 0.066). There was a trend toward lower activity in the control HU animals irradiated with 1.5 Gy than those irradiated with 0.75 Gy that did not reach significance (*p* = 0.0959, Dunnett’s test). There was also a day effect (*F* (1, 123) = 136.269, *p* < 0.001), with higher activity levels on day 4 for one familiar and one novel object than day 3 for two identical objects, indicating that the animals responded to the novelty.

The animals spent relatively little time exploring the objects on day 4 (Figure 3B). There was no effect of radiation or HU condition. When rats that did not explore the objects on a day were excluded from the analysis, there was no significant effect of the condition (*F* (2, 54) = 2.813, *p* = 0.0689) but animals under HU spent more time exploring the objects than animals without HU (*p* = 0.0464, Dunnett’s test, Figure 3C). When the percentage of animals that explored the objects on day 4 was examined, the animals under HU showed a trend toward a lower percentage (27%) than animals without HU (48%) (*z* (1, 3.805) = 1.951, *p* = 0.051, two-sided chi-squared test) or animals under the control HU condition (50%) (*z* (1, 3.732) = 1.932, *p* = 0.053, two-sided chi-squared test).

### 3.3 Plasma metabolomics analysis

**FIGURE 4:**
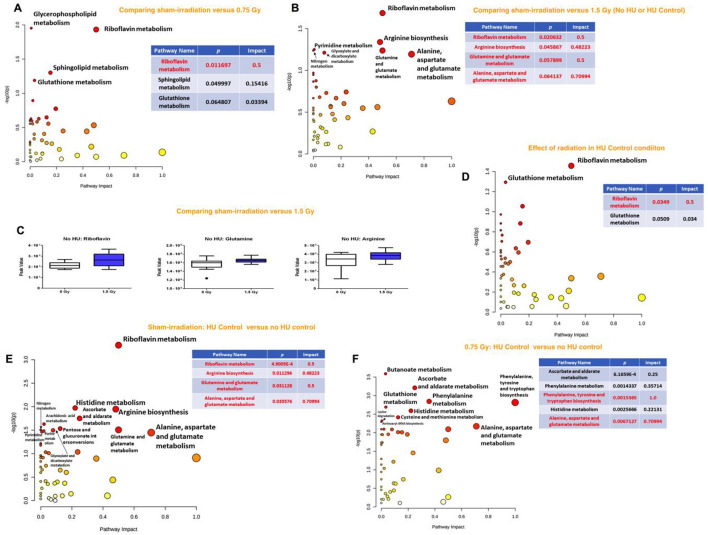
**(A)** Analysis of the effects of radiation in the plasma of animals without HU or control HU showed that the riboflavin metabolic pathway was most affected with the glycerophospholipid, sphingolipid, and glutathione metabolic pathways being less affected for sham irradiation vs. 0.75 Gy exposure. **(B)** Sham irradiation vs. 1.5 Gy exposure in animals without HU or control HU showed that the riboflavin metabolic pathway was affected most along with the arginine biosynthesis, glutamine and glutamate metabolic, and alanine, aspartate, and glutamate metabolic pathways. **(C)** Riboflavin, glutamine, and arginine levels were higher in the plasma of animals irradiated with 1.5 Gy than the sham-irradiated animals. **(D)** In the plasma of animals without control HU, the riboflavin metabolic pathway was the most affected, while the glutathione metabolic pathway was less affected. **(E)** In the assessment of the effects of the control HU condition on plasma in sham-irradiated animals, the alanine, aspartate, and glutamate metabolic, riboflavin metabolic, arginine biosynthesis, and glutamine and glutamate metabolic pathways were found to be affected. **(F)** In the animals exposed to 0.75 Gy, assessment of the effects of the control HU condition on plasma showed that the phenylalanine, tyrosine, and tryptophan pathway was most affected, followed by strong effects on the alanine, aspartate, and glutamate metabolic pathways. The metabolome view figures contain all the matched pathways (metabolomes) arranged by *p*-values (generated as part of the pathway enrichment analysis) along the *Y* axis and pathway impact values (generated as part of the pathway topology analysis) along the *X* axis. The node colors are based on the *p* values, and the node radii are determined on the basis of the pathway impact values. The red nodes indicate strong impact and significance, while the yellow nodes indicate weak impact and non-significant *p*-value. The pathway impact and *p*-values are indicated in the tables.

When the effects of radiation were analyzed in the plasma of animals without HU or control HU, the riboflavin metabolic pathway was affected most along with glycerophospholipid, sphingolipid, and glutathione metabolic pathways being less affected in the comparison of sham irradiation vs. 0.75 Gy (Figure 4A). Comparison of sham irradiation vs. 1.5 Gy irradiation in animals without HU or control HU showed that the riboflavin metabolic pathway was also affected (Figure 4B). The arginine biosynthesis; glutamine and glutamate metabolism; and alanine, aspartate, and glutamate metabolic pathways were also affected (Figure 3B). Riboflavin, glutamine, and arginine concentrations were higher in the plasma of animals that received 1.5 Gy irradiation than the sham-irradiated animals (Figure 4C).

When the effects of radiation were analyzed in the plasma of animals under the control HU condition, the riboflavin metabolic pathway was maximally affected, while the glutathione metabolic pathway was affected to a lesser extent (Figure 4D). In contrast, none of the pathways were significantly affected when comparing the 1.5 Gy vs. sham-irradiation animals.

When comparing the effects of the control HU condition on plasma in the sham-irradiated animals with those not under the condition, the alanine, aspartate, and glutamate metabolism, as well as riboflavin metabolism, arginine biosynthesis, and glutamine and glutamate metabolism were found to be affected (Figure 4E). Next, in the 0.75-Gy-irradiated animals, the effects of the control HU condition on plasma were analyzed by comparison with those not under the condition. The phenylalanine, tyrosine, and tryptophan pathway was the most affected one but the alanine, aspartate, and glutamate metabolic pathway was also strongly affected (Figure 4F). In contrast, none of the pathways were found to be significantly affected when assessing the effects of the control HU condition on 1.5-Gy-irradiated animals.

### 3.4 Metabolomics analysis of the hippocampus

When the effects of radiation were analyzed in the hippocampi of animals without HU or control HU, none of the pathways were found to be affected in animals receiving 0.75 Gy of radiation and none of the pathways had impacts of 0.5 or higher in animals receiving 1.5 Gy. The purine metabolism and pyrimidine pathway was affected and had impacts of 0.28 and 0.23, respectively (Supplementary Figure S1).

**FIGURE 5:**
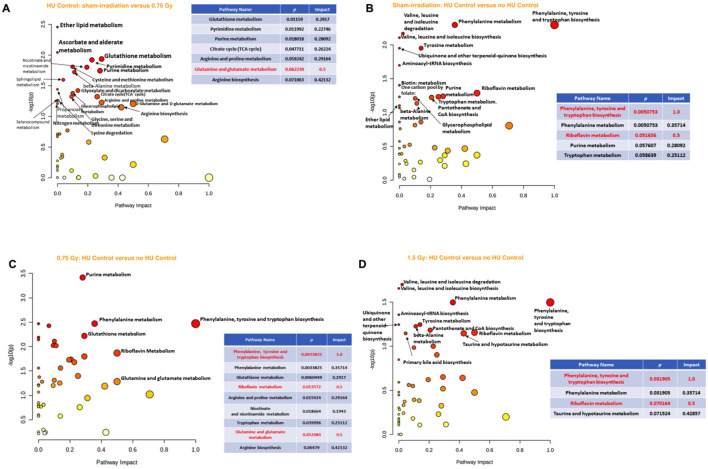
**(A)** Analysis of the effects of 0.75 Gy irradiation in the hippocampus of animals under the control HU condition showed that the glutamine and glutamate metabolic pathway was affected. **(B)** In sham-irradiated animals, assessment of the effects of the control HU condition on the hippocampus showed that the phenylalanine, tyrosine, and tryptophan pathway was most affected, followed by the riboflavin metabolic pathway. **(C)** In animals exposed to 0.75 Gy, the phenylalanine, tyrosine, and tryptophan pathway was the most affected, followed by the riboflavin metabolic as well as D-glutamine and D-glutamate pathways. **(D)** In animals exposed to 1.5 Gy, the phenylalanine, tyrosine, and tryptophan pathway was the most affected, followed by the riboflavin metabolic pathway. The red nodes indicate strong impact and significance, while the yellow nodes indicate weak impact and a non-significant *p-*value. The pathway impact and *p*-values are indicated in the tables.

When the effects of exposure to 0.75 Gy of irradiation were analyzed in the hippocampi of animals under the control HU condition, the glutamine and glutamate metabolic pathway was found to be affected (Figure 5A). In contrast, when the effects of exposure to 1.5 Gy of irradiation were analyzed in the hippocampi of animals under the control HU condition, only fatty acid biosynthesis was identified, with an impact of 0.015 (Supplementary Figure S2).

In the sham-irradiated animals, the effects on the hippocampus were compared in animals under and not under the control HU condition, and it was found that the phenylalanine, tyrosine, and tryptophan pathway was affected most followed by the riboflavin metabolic pathway (Figure 5B). In the 0.75-Gy-irradiated animals, the phenylalanine, tyrosine, and tryptophan pathway was also affected the most, followed by the riboflavin metabolic as well as D-glutamine and D-glutamate pathways (Figure 5C). In the 1.5-Gy-irradiated animals, the phenylalanine, tyrosine, and tryptophan pathway was also the most affected, followed by the riboflavin metabolic pathway (Figure 5D).

### 3.5 Metabolomics analysis of the cortex

When the effects of radiation were analyzed in the cortixes of animals not under the HU or control HU condition, there were no pathways that had impacts of 0.5 or greater in animals irradiated with 0.75 Gy (Supplementary Figure S3A) or 1.5 Gy (Supplementary Figure S3B).

**FIGURE 6:**
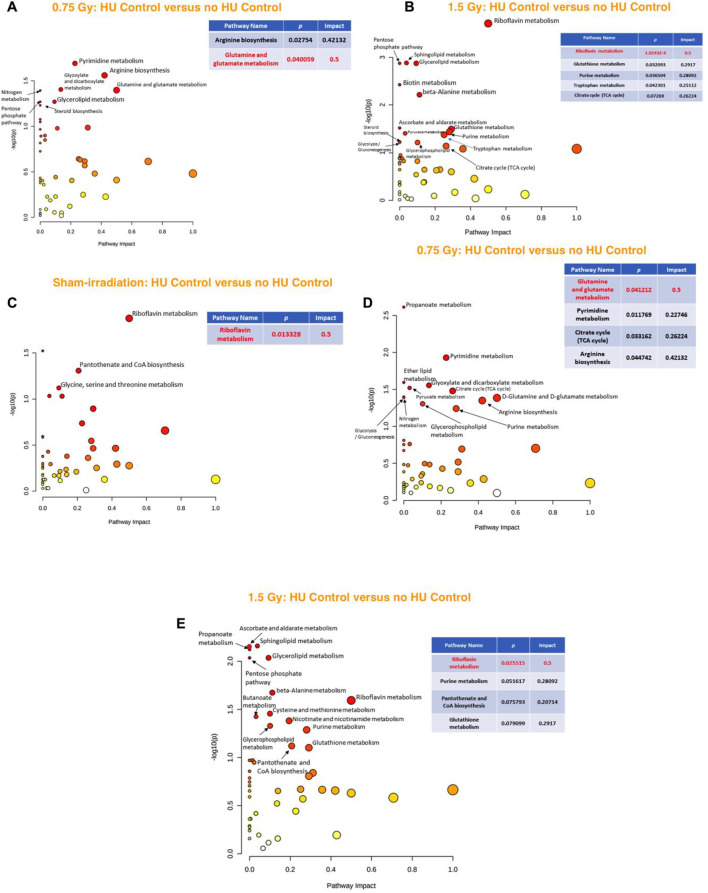
**(A)** Analysis of the effects of 0.75 Gy irradiation in the cortixes of animals under the control HU condition showed that the glutamine and glutamate metabolic pathway was affected. **(B)** Assessment of the effects of 1.5 Gy irradiation in the cortixes of animals under the control HU condition showed that the riboflavin pathway was affected. **(C)** In sham-irradiated animals, assessment of the effects of the control HU condition on the cortex showed that the riboflavin metabolic pathway was affected. **(D)** In animals exposed to 0.75 Gy, the glutamine and glutamate metabolic pathway was affected. **(E)** In animals exposed to 1.5 Gy, the riboflavin metabolic pathway was affected. The red nodes indicate strong impact and significance, while the yellow nodes indicate weak impact and non-significant *p*-value. The pathway impact and *p*-values are indicated in the tables.

When the effects of exposure to 0.75 Gy of irradiation were analyzed in the cortixes of animals under the control HU condition, the glutamine and glutamate metabolism pathway was found to be affected, similar to that of the hippocampus (Figure 6A). Next, exposure to 1.5 Gy of irradiation on the cortixes of animals under the control HU condition showed that the riboflavin pathway was affected (Figure 6B).

In the sham-irradiated animals, the cortical effects were analyzed by comparing animals under and not under the control HU condition, which showed that the riboflavin metabolic pathway was affected (Figure 6C). In the 0.75-Gy-irradiated animals, the glutamine and glutamate metabolic pathway was affected (Figure 6D), while the riboflavin metabolic pathway was affected in the 1.5-Gy-irradiated animals (Figure 6E).

### 3.6 Individual metabolites in the affected pathways in the plasma, hippocampus, and cortex

**FIGURE 7:**
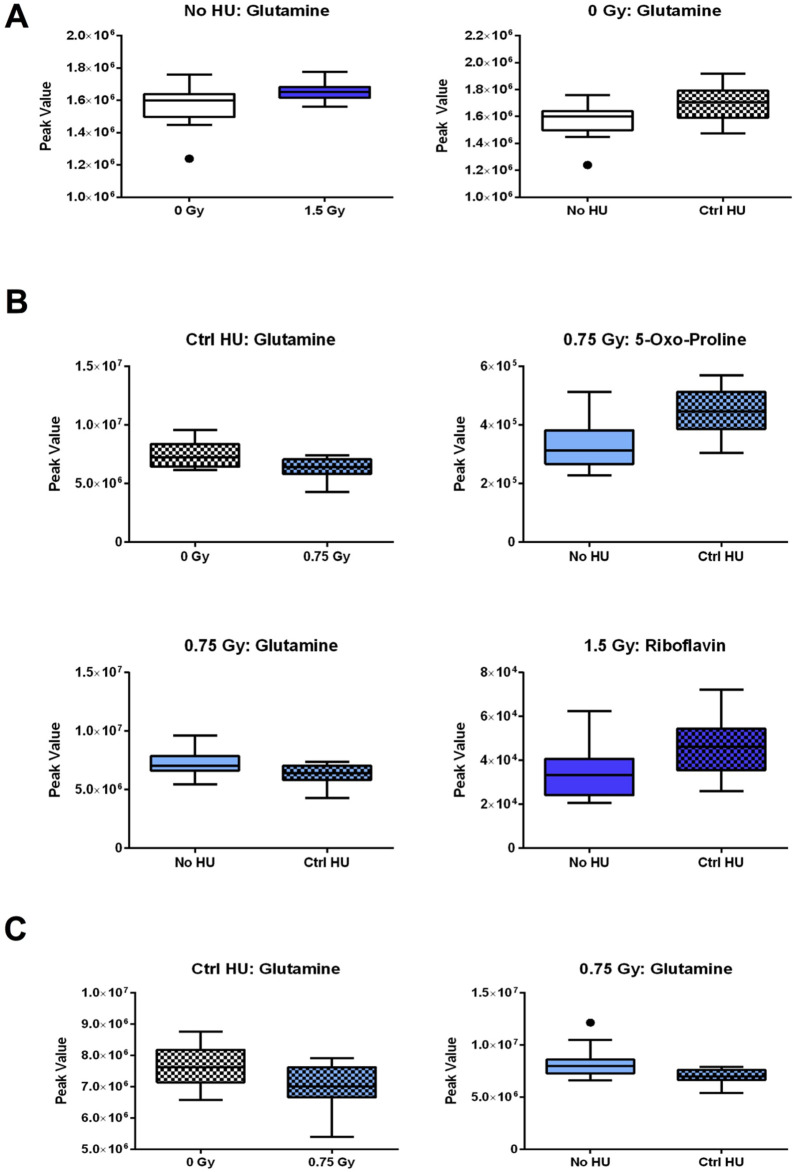
Individual metabolites in the glutamine and glutamate pathway. **(A)** In the plasma of animals without HU, the glutamine levels were higher for 1.5 Gy exposure than sham irradiation; the glutamine levels were also higher in the sham-irradiated animals in the control HU condition than those without HU. In the **(B)** hippocampi and **(C)** cortixes of animals in the control HU condition, the glutamine levels were lower for exposure to 0.75 Gy than sham irradiation; furthermore, the glutamine levels were lower in animals in the control HU condition than those without HU. **(B)** In the hippocampi of animals exposed to 0.75 Gy, the 5-oxo-proline levels were higher under HU than without HU. In the hippocampi of animals exposed to 1.5 Gy, the riboflavin levels were higher under the control HU condition than without HU. The red nodes indicate strong impact and significance, while the yellow nodes indicate weak impact and non-significant *p-*value. The pathway impact and *p*-values are indicated in the tables.

The individual metabolites in the glutamine and glutamate pathway are shown in Figure 7. In the plasma of animals without HU, the glutamine levels were higher in animals exposed to 1.5 Gy irradiation than the sham-irradiated animals (Figure 7A); these levels were also higher in sham-irradiated animals in the control HU condition than those without HU (Figure 7A). In the hippocampi (Figure 7B) and cortixes (Figure 7C) of animals under the control HU condition, the glutamine levels were lower for exposure to 0.75 Gy than in the sham-irradiated animals. In the 0.75-Gy-irradiated animals, the glutamine levels were lower in the hippocampi (Figure 7B) and cortixes (Figure 7C) under the control HU condition than without HU. In addition, in the hippocampi of animals exposed to 0.75 Gy of radiation, the levels of 4/5-oxo-proline were higher in animals under HU than those without HU. In the hippocampi of animals exposed to 1.5 Gy of radiation, the riboflavin levels were higher in animals under the control HU condition than those without HU (Figure 7B).

**FIGURE 8:**
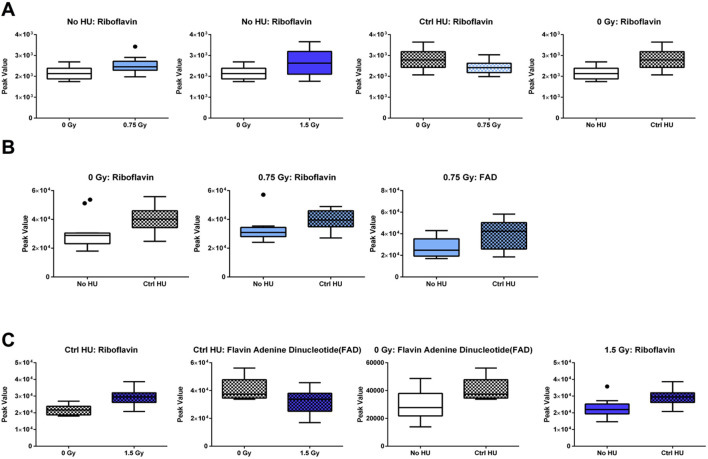
Individual metabolites in the riboflavin metabolic pathway. **(A)** In the plasma of animals without HU, the riboflavin levels were higher for exposure to 0.75 or 1.5 Gy than sham irradiation; the riboflavin levels were also higher in the plasma of sham-irradiated animals under the control HU condition than those without HU. In the plasma of animals under the HU condition, the riboflavin levels were lower for 0.75 Gy exposure than sham irradiation. **(B)** In the hippocampi of animals receiving sham or 0.75 Gy irradiation, the riboflavin levels were higher under the control HU condition than without HU. In the 0.75-Gy-irradiated animals, FAD levels were higher in the **(B)** hippocampus and **(C)** cortex under HU than without HU. In the cortixes of animals under the control HU condition, the riboflavin levels were higher and FAD levels were lower for 1.5 Gy exposure than sham irradiation. In the 1.5-Gy-irradiated animals, the riboflavin levels were higher under the control HU condition than without HU. The red nodes indicate strong impact and significance, while the yellow nodes indicate weak impact and a non-significant *p*-value. The pathway impact and *p-*values are indicated in the tables. The box plots help with identifying outliers based on data points with values beyond the whiskers (indicated as black circles).

The individual metabolites in the riboflavin metabolic pathway are indicated in Figure 8. In the plasma of animals without HU, the riboflavin levels were higher when exposed to 0.75 Gy or 1.5 Gy of radiation than the sham-irradiated animals (Figure 8A). The riboflavin levels were also higher in the plasma of sham-irradiated animals under the control HU condition than those without HU. In the plasma of animals under the HU condition, the riboflavin levels were lower when exposed to 0.75 Gy of radiation than in the sham-irradiated animals. In the hippocampi of sham-irradiated and 0.75-Gy-irradiated animals, the riboflavin levels were higher under the control HU condition than those without HU (Figure 8B). In the 0.75-Gy-irradiated animals, the flavin adenine dinucleotide (FAD) levels were higher in the hippocampi (Figure 8B) and cortixes (Figure 8C) of animals under HU than without HU. In the cortixes of animals under the control HU condition, the riboflavin levels were higher and FAD levels lower when exposed to 1.5 Gy of radiation than sham irradiation.

**FIGURE 9:**
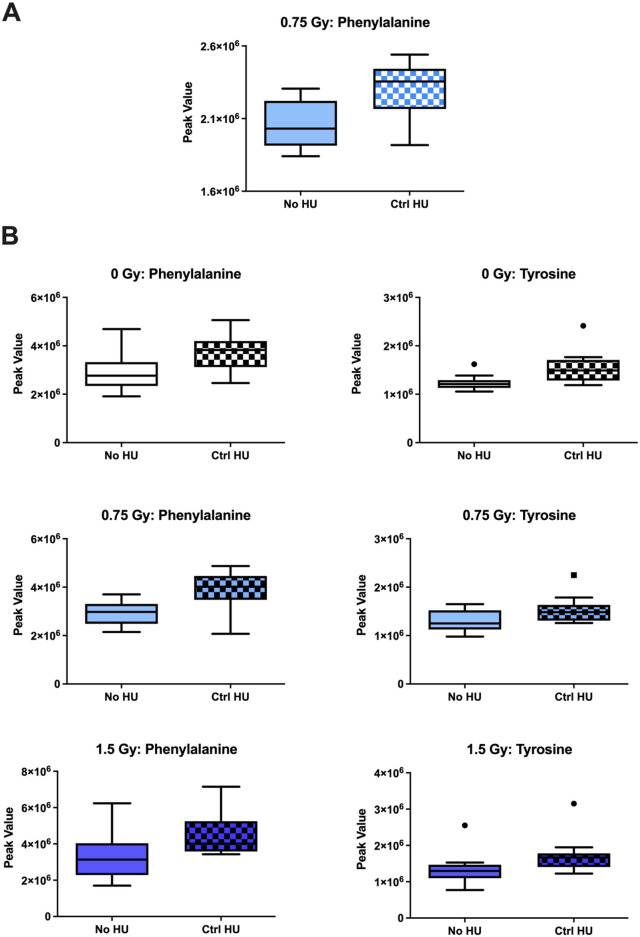
Individual metabolites in the phenylalanine, tyrosine, and tryptophan biosynthesis pathway. **(A)** In the plasma of animals exposed to 0.75 Gy, the phenylalanine levels were higher under HU than without HU. **(B)** Tyrosine and phenylalanine levels were higher in the hippocampi of the animals receiving sham, 0.75 Gy, and 1.5 Gy irradiation. The box plots help with identifying outliers based on data points with values beyond the whiskers (indicated as black circles).

The individual metabolites in the phenylalanine, tyrosine, and tryptophan biosynthesis pathway are shown in Figure 9. In the plasma of animals exposed to 0.75 Gy of radiation, phenylalanine levels were higher under the HU condition than without HU (Figure 9A); tyrosine and phenylalanine were higher in the hippocampi of the sham-, 0.75-Gy-, and 1.5-Gy-irradiated animals (Figure 9B).

**FIGURE 10:**
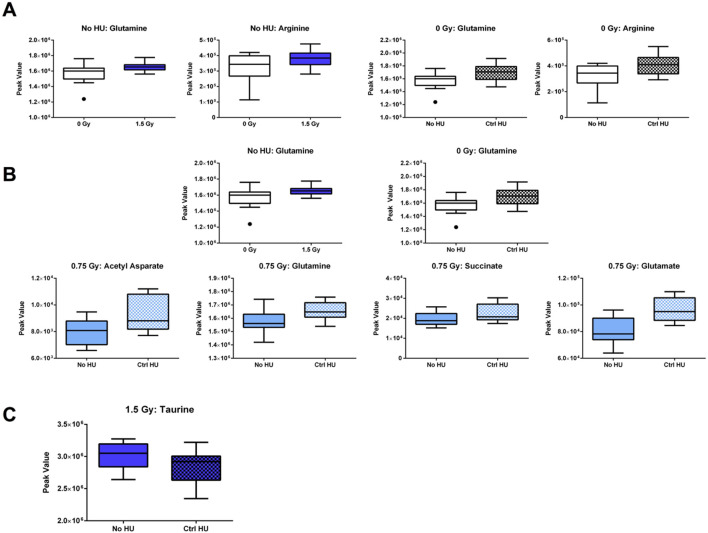
**(A)** Individual metabolites in the arginine biosynthesis pathway. In animals without HU or under the control HU condition, glutamine and arginine levels were higher for exposure to 1.5 Gy than sham irradiation; glutamine and arginine levels were also higher in the plasma of sham-irradiated animals under the control HU condition than those without HU. **(B)** Individual metabolites in the alanine, aspartate, and glutamate metabolic pathway. In the plasma of animals without HU or under the control HU condition, the glutamine levels were higher for 1.5 Gy exposure than sham irradiation. In animals exposed to sham irradiation and 0.75 Gy, the glutamine levels were higher under the control HU condition than without HU. In the plasma of 0.75-Gy-irradiated animals, acetyl aspartate, succinate, and glutamate were higher under the control HU condition than without HU. **(C)** In the primary bile acid synthesis pathway, taurine levels in the hippocampi of 1.5-Gy-irradiated animals under the HU condition were lower than those in animals without HU or control HU. The box plots help with identifying outliers based on data points with values beyond the whiskers (indicated as black circles).

The individual metabolites in the arginine biosynthesis pathway are indicated in Figure 10. In animals without HU or control HU, glutamine and arginine levels were higher when exposed to 1.5 Gy radiation than sham irradiation (Figure 10A). In addition, glutamine and arginine levels were higher in the plasma of sham-irradiated animals under the control HU condition than those without HU (Figure 10A).

 The individual metabolites in the alanine, aspartate, and glutamate metabolic pathway are indicated in Figure 10B. In the plasma of animals without HU or control HU, glutamine levels were higher upon exposure to 1.5 Gy than sham irradiation (Figure 10B). In addition, in the sham- and 0.75-Gy-irradiated animals, glutamine levels were higher under the control HU condition than without HU (Figure 10B). In the plasma of 0.75-Gy-irradiated animals, acetyl aspartate, succinate, and glutamate were also higher under the control HU condition than without HU. Finally, in the primary bile acid synthesis pathway, taurine levels in the hippocampi of animals exposed to 1.5 Gy irradiation under the HU condition were lower than those without HU or control HU (Figure 10C).

### 3.7 Regression analysis of individual metabolites and specific behavioral or cognitive measures

We used univariate linear regression analyses stratified by radiation exposure and HU condition along with Z-scores for the behavioral measures (dependent variables) and metabolite values (independent variables) to identify potential plasma biomarkers of radiation exposure or HU condition on the behavioral or cognitive performance. We selected only those metabolites that were consistently included in the models. Table 2 lists all the behavioral measures used in the regression analysis in this study.

Table 3 illustrates the 21 plasma metabolites that were consistently included in the models (at least four times each) and the 10 behavioral measures related to them. Twelve of the associations between the metabolites and behavioral measures were purely positive, as indicated in green font in Table 3. One of the metabolites was purely negatively associated with the behavioral measures (aspartate), as indicated in red font in Table 3. The remaining eight metabolites showed both positive and negative associations with the behavioral measures, as indicated in blue font in Table 3.

**TABLE 2:** Behavioral measures included for regression analyses with individual metabolites^a^.

Measure	Definition
Activity	Distance moved in the open field on the first day of testing
Activity	Distance moved in the open field on the second day of testing
Activity	Distance moved in the object recognition training trial
Activity	Distance moved in the object recognition test trial
Anxiety	Percentage of time spent in the center of the open field on the first day of testing
Anxiety	Percentage of time spent in the center of the open field on the second day of testing
Cognition	Total time spent exploring the objects in the object recognition test
Cognition	Percentage of time spent in the center of the open field during the object recognition training trial
Cognition	Percentage of time spent in the center of the open field during the object recognition test trial
Cognition	Discrimination index for the object recognition test

^a^ The same measures were used in the current study and our earlier study on Fischer rats with the following three exceptions: 1) in the previous study, a measure of anhedonia was included; 2) in the previous study, a measure of anxiety in the elevated plus maze was included; 3) in the previous study, the total time spent exploring during the object recognition training trial was included.

**TABLE 3:** Associations of specific metabolites with the behavioral measures^b^.

Condition	Dose (Gy)	Compartment	Metabolite^b^	*N* ^b^	Significant positive associations	Significant negative associations
No HU	0	Cortex	2-Hydroxy-C18-cerebroside	4	Percentage of time spent in the center of the open field during the object recognition training trial; distance moved in the object recognition training trial: distance moved in the object recognition test trial	Discrimination index for the object recognition test
No HU	0	Cortex	Lauroylcarnitine	4	Total time spent exploring the objects in the object recognition test; percentage of time spent in the center of the open field during the object recognition test trial; distance moved in the object recognition test trial; percentage of time spent in the center of the open field during the object recognition test trial	
No HU	0	Cortex	Sphingosine-1-phosphate	5	Total time spent exploring the objects in the object recognition test; percentage of time spent in the center of the open field during the object recognition test trial; distance moved in the object recognition test trial; percentage of time spent in the center of the open field in the object recognition test trial; distance moved in the object recognition test trial	
No HU	0.75	Cortex	2-Aminoethyl dihydrogen phosphate	4	Percentage of time spent in the center of the open field during the object recognition training trial; distance moved in the object recognition test trial	Total time spent exploring the objects in the object recognition test; percentage of time spent in the center of the open field in the object recognition test trial
No HU	0.75	Cortex	Hypoxanthine	4	Percentage of time spent in the center of the open field on the first day of testing	Total time spent exploring the objects in the object recognition test; percentage of time spent in the center of the open field in the object recognition test trial; distance moved in the object recognition test trial
No HU	0.75	Cortex	Phenylalanine	4	Percentage of time spent in the center of the open field on the first day of testing	Total time spent exploring the objects in the object recognition test; percentage of time spent in the center of the open field in the object recognition test trial; distance moved in the object recognition test trial
No HU	0.75	Cortex	Sphingomyelin	4	Percentage of time spent in the center of the open field during the object recognition training trial; distance moved in the open field on the second day of testing	Total time spent exploring the objects in the object recognition test; percentage of time spent in the center of the open field during the object recognition test trial
No HU	0.75	Cortex	Tryptophan	4	Percentage of time spent in the center of the open field on the first day of testing	Total time spent exploring the objects in the object recognition test; percentage of time spent in the center of the open field during the object recognition test trial; distance moved in the object recognition test trial
No HU	0.75	Cortex	Valine	4	Percentage of time spent in the center of the open field on the first day of testing	Total time spent exploring the objects in the object recognition test; percentage of time spent in the center of the open field in the object recognition test trial; distance moved in the object recognition test trial
Ndo HU	0.75	Hippocampus	Adenine	4	Discrimination index for the object recognition test	Percentage of time spent in the center of the open field in the object recognition training trial; distance moved in the object recognition training trial; distance moved in the open field on the second day of testing
No HU	1.5	Hippocampus	Sodium benzoate	5	Percentage of time spent in the open field in the object recognition training trial; distance moved in the object recognition training trial; percentage of time spent in the center of the open field on the first day of testing; distance moved in the open field on the first day of testing; distance moved in the open field on the second day of testing	
No HU	1.5	Plasma	Deoxycarnitine	4	Discrimination index for the object recognition test; percentage of time spent in the center of the open field in the object recognition test trial; distance moved in the open field on the first day of testing; distance moved in the open field on the second day of testing	
No HU	1.5	Plasma	Sphingomyelin	4	Discrimination index for the object recognition test; total time spent exploring the objects in the object recognition test; percentage of time spent in the center of the open field in the object recognition test trial; distance moved in the object recognition test trial	
Control HU	0.75	Hippocampus	Succinate/methylmalonate	4	Percentage of time spent in the center of the open field during the object recognition training trial; percentage of time spent in the open field during the object recognition test trial; distance moved in the open field on the first day of testing	
Control HU	0.75	Plasma	4-Guanidinobutanoic acid	4	Percentage of time spent in the center of the open field in the object recognition training trial; distance moved in the object recognition training trial; percentage of time spent in the center of the open field on the first day of testing; distance moved in the open field on the first day of testing	
Control HU	0.75	Plasma	5-Methylcytosine	4	Percentage of time spent in the center of the open field in the object recognition training trial; distance moved in the object recognition training trial; percentage of time spent in the center of the open field on the first day of testing; distance moved in the open field on the first day of testing	
Control HU	0.75	Plasma	Citrulline	4	Percentage of time spent in the center of the open field in the object recognition training trial; distance moved in the object recognition training trial; percentage of time spent in the center of the open field on the first day of testing; distance moved in the open field on the first day of testing	
Control HU	0.75	Plasma	Lauroylcarnitine	4	Percentage of time spent in the center of the open field in the object recognition training trial; distance moved in the object recognition training trial; percentage of time spent in the center of the open field on the first day of testing; distance moved in the open field on the first day of testing	
Control HU	0.75	Plasma	Pipecolic acid	4	Percentage of time spent in the center of the open field in the object recognition training trial; distance moved in the object recognition training trial; percentage of time spent in the center of the open field on the first day of testing; distance moved in the open field on the first day of testing	
Control HU	0.75	Plasma	Protoporphyrin	5	Percentage of time spent in the center of the open field in the object recognition training trial; percentage of time spent in the center of the open field in the object recognition test trial; distance moved in the object recognition test trial; percentage of time spent in the center of the open field on the first day of testing; distance moved in the open field on the first day of testing	
Control HU	1.5	Hippocampus	Aspartate	5		Total time spent exploring the objects in the object recognition test; percentage of time spent in the center of the open field during the object recognition training trial; percentage of time spent in the center of the open field in the object recognition test trial

^a^ Metabolites that are purely positively associated with behavioral measures are indicated in green; metabolites that are purely negatively associated with behavioral measures are indicated in red; metabolites that are both positively and negatively associated with behavioral measures are indicated in blue.

^b^ N indicates the number of significant associations.

In the cortixes of the sham-irradiated animals without HU, two metabolites were purely positively correlated and one metabolite was both positively and negatively correlated with the behavioral measures.

In animals under the control HU condition exposed to 0.75 Gy of radiation, seven metabolites were purely positively correlated with the behavioral measures (one in the hippocampus and six in the plasma).

In contrast, in animals without HU that were exposed to 0.75 Gy of radiation, seven metabolites that were both positively and negatively correlated with the behavioral measures (six in the cortex and one in the hippocampus).

In the control HU animals exposed to 1.5 Gy of radiation, one metabolite in the hippocampus was purely negatively correlated with the behavioral measures. In contrast, in animals without HU exposed to 1.5 Gy of radiation, three metabolites were purely positively correlated with the behavioral measures (one in the hippocampus and two in the plasma).

We next assessed whether any of the 21 metabolites identified in the present study were also among the 27 metabolites in the plasma of photon-irradiated Fischer rats in our previous study 9 months after exposure (Raber et al., 2024). Succinate and 5-methylcytosine were the two metabolites identified in both studies that showed positive associations with the behavioral measures of both studies (Table 4). In the current study, succinate in the hippocampus and 5-methylcytosine in the plasma of the control HU rats exposed to 0.75 Gy of radiation were associated with the behavioral measures. In our previous study in photon-irradiated rats, only the plasma metabolites were analyzed. Succinate in the sham-irradiated rats without HU and 5-methylcytosine in rats exposed to 8 Gy without HU were associated with the behavioral measures. Regarding the behavioral measures, the percentage of time spent in the field was correlated with succinate levels in both studies. For 5-methylcytosine, there was no overlap in the behavioral measures identified to be correlated in both studies. However, the common behavioral measures used in both studies were for object recognition. In the present study, correlations were observed with the behavioral measures on the second day of the object recognition test; however, in the photon irradiation study, correlations with the behavioral measures were observed on the first day of the object recognition test.

**TABLE 4:** Metabolites shown to be correlated with behavioral measures in our previous photon-irradiated animals^a^ that were also found to be associated with the behavioral measures in the present study.

Non-HU/HU	Dose (Gy)	Metabolite^a^	N^b^	Significant positive associations	Significant negative associations
No HU	0	Succinate	7	Ratio of time spent in the open arms in the elevated plus maze; percentage of time spent in the center of the open field in the object recognition test trial; distance moved in the object recognition test trial; total time spent exploring the objects in the object recognition test; percentage of time spent in the center of the open field on the first day of testing; percentage of time spent in the center of the open field on the second day of testing	
No HU	8	5-Methylcytosine	5	Percentage of M&M consumed on days 2 and 3 compared to day 1; percentage of time spent in the center of the open field in the object recognition test trial; discrimination index for the object recognition test; distance moved in the object recognition test trial; total time spent exploring the objects in the object recognition test	

^a^ Plasma metabolites positively associated with behavioral measures are indicated in green.

^b^ N indicates the number of significant associations.

Next, we used the same univariate linear regression analyses stratified by radiation exposure and HU condition with the Z-scores for behavioral measures (dependent variables) and metabolite values (independent variables) to identify potential plasma biomarkers of radiation exposure or HU condition on the behavioral or cognitive performances in our earlier study with WAG/Rij rats (Raber et al., 2021). Male WAG/Rij rats were behaviorally and cognitively tested 3 months after exposure to sham irradiation or simGCRsim in the absence or presence of HU alone. Six months following the behavioral and cognitive tests or 9 months following sham or total body irradiation, the plasma and brain tissues (hippocampus and cortex) were processed to determine whether the behavioral and cognitive effects were associated with long-term alterations in the metabolic pathways in the plasma and brain. We selected only those metabolites that were consistently included in the models. Table 5 illustrates 40 metabolites that were consistently included in the models (at least four times each) and the behavioral measures related to them. Of these 40 metabolites, 15 were present in the cortex, 4 were identified in the hippocampus, and 21 were observed in the plasma.

Eighteen associations between the metabolites and behavioral measures were purely positive, as indicated in green font in Table 5; seven metabolites were purely negatively associated with the behavioral measures, as indicated in red font in Table 5; and fifteen metabolites showed both positive and negative associations with the behavioral measures, as indicated in blue font in Table 5.

**TABLE 5:** Associations of specific metabolites with the behavioral measures^b^.

Condition	Dose (Gy)	Compartment	Metabolite^b^	N^b^	Significant positive associations	Significant negative associations
No HU	0	Hippocampus	N(pai)-methyl-l-histidine	6		Total time spent exploring the objects in the training trial; total time spent exploring the objects in the test trial; distance moved in the object recognition training trial; distance moved in the object recognition test trial; distance moved in the open field on the first day of testing; distance moved in the open field on the second day of testing
No HU	0	Hippocampus	Phenylalanine	4	Total time spent exploring the objects in the test trial; distance moved in the object recognition test trial; distance moved in the open field on the second day of testing	Percentage immobility in the forced swim test
No HU	0	Hippocampus	Tryptophan	4	Total time spent exploring the objects in the test trial; distance moved in the object recognition test trial; distance moved in the open field on the second day of testing	Percentage immobility in the forced swim test
No HU	0	Plasma	4-Acetamidobutanoate	4	Total time spent exploring the objects in the test trial; distance moved in the object recognition training trial; distance moved in the open field on the second day of testing	Percentage immobility in the forced swim test
No HU	0	Plasma	N-Acetyl-L-phenylalanine	4	Total time spent exploring the objects in the test trial; distance moved in the object recognition test trial; distance moved in the open field on the first day of testing; distance moved in the open field on the second day of testing	
No HU	1.5	Cortex	3-Methylhistamine	4	Total time spent exploring the objects in the habituation trial; distance moved in the habituation trial; distance moved in the open field on the second day of testing; percentage of time spent in the center of the open field on the second day of testing	
No HU	1.5	Cortex	Cytidine	5	Total time spent exploring the objects in the acquisition trial; total time spent exploring the objects in the training trial; distance moved in the object recognition training trial; distance moved in the open field on the first day of testing; distance moved in the open field on the second day of testing	
No HU	1.5	Cortex	Elaidic acid	4	Percentage of time spent in the center of the open field during the object recognition acquisition trial; percentage of time spent in the center of the open field in the object recognition training trial; distance moved in the object recognition acquisition trial; distance moved in the object recognition training trial	
No HU	1.5	Cortex	Heptadecanoate	4	Total time spent exploring the objects in the acquisition trial; total time spent exploring the objects in the training trial; distance moved in the object recognition training trial; distance moved in the open field on the second day of testing	
No HU	1.5	Cortex	Histamine	5	Total time spent exploring the objects in the acquisition trial; total time spent exploring the objects in the training trial; distance moved in the object recognition acquisition trial; distance moved in the object recognition training trial; distance moved in the object recognition test trial; distance moved in the open field on the second day of testing	
No HU	1.5	Cortex	Inosine	5	Total time spent exploring the objects in the acquisition trial; total time spent exploring the objects in the training trial; distance moved in the object recognition acquisition trial; distance moved in the object recognition training trial; distance moved in the object recognition test trial; distance moved in the open field on the first day of testing; distance moved in the open field on the second day of testing	
No HU	1.5	Cortex	Kynurenine	4	Total time spent exploring the objects in the training trial; Percentage of time spent in the center of the open field in the object recognition training trial; total distance in the object recognition acquisition trial; distance moved in the open field on the first day of testing	
No HU	1.5	Cortex	Myristic acid	4	Total time spent exploring the objects in the object recognition training trial; distance moved in the object recognition acquisition trial; distance moved in the object recognition training trial; distance moved in the open field on the second day of testing	
No HU	1.5	Cortex	Palmitate	5	Total time spent exploring the objects in the training trial; time spent in the center of the open field in the object recognition training trial; distance moved in the object recognition acquisition trial; percentage of time spent in the center of the open field on the second day of testing; distance moved in the open field on the second day of testing	
No HU	1.5	Cortex	Palmitoleic acid	4	Total time spent exploring the objects in the object recognition acquisition trial; total time spent exploring the objects in the object recognition training trial; distance moved in the object recognition acquisition trial; distance moved in the object recognition training trial	
No HU	1.5	Cortex	Pantothenic acid	5	Total time spent exploring the objects in the object recognition acquisition trial; total time spent exploring the objects in the object recognition training trial; distance moved in the object recognition acquisition trial; distance moved in the open field on the first day of testing; distance moved in the open field on the second day of testing	
No HU	1.5	Hippocampus	Fructose-1,6-biphosphate	4	Total time spent exploring the objects in the object recognition acquisition trial; total time spent exploring the objects in the object recognition training trial; percentage of time spent exploring the novel object in the object recognition test trial; percentage of time spent in the center of the open field on the first day of testing	
Npo HU	1.5	Plasma	Carnitine	4	Total time spent exploring the objects in the test trial; percentage of time spent exploring the familiar object in the object recognition test trial	Total time spent exploring the objects in the object recognition acquisition trial; percentage of time spent in the center of the open field in the object recognition acquisition trial
No HU	1.5	Plasma	Carnosine	4	Time spent exploring objects in the object recognition test trial; percentage of time spent exploring the familiar object in the object recognition test trial; percentage immobility in the forced swim test	Distance moved in the object recognition acquisition trial
No HU	1.5	Plasma	Elaidic acid	4	Distance moved in the object recognition training trial; distance moved in the object recognition test trial; distance moved in the open field on the second day of testing	Percentage immobility in the forced swim test
No HU	1.5	Plasma	Glucosamine	4	Percentage of time spent in the open field on the second day of testing; percentage of time spent in the center of the open field in the object recognition test trial; distance moved in the object recognition acquisition trial; distance moved in the object recognition test trial	
No HU	1.5	Plasma	Glucose	4	Percentage of time spent in the center of the open field on the second day of testing; distance moved in the open field on the second day of testing; distance moved in the object recognition acquisition trial; distance moved in the object recognition test trial	
Ndo HU	1.5	Plasma	Methyl-indole-3-acetate	5	Percentage of time spent in the center of the open field on the second day of testing; distance moved in the open field on the second day of testing; total time spent exploring the objects in the object recognition training trial; distance moved in the object recognition acquisition trial; distance moved in the object recognition test trial	
No HU	1.5	Plasma	Myristic acid	4	Percentage of time spent in the center of the open field during the object recognition test trial; distance moved in the object recognition acquisition trial; distance moved in the object recognition test trial	Percentage immobility in the forced swim test
No HU	1.5	Plasma	Nonanoate	4	Distance moved in the object recognition acquisition trial; distance moved in the object recognition test trial; distance moved in the open field on the second day of testing	Percentage immobility in the forced swim test
No HU	1.5	Plasma	Palmitate	4	Percentage of time spent in the center of the open field in the object recognition test trial; distance moved in the open field in the object recognition acquisition trial; distance moved in the object recognition test trial	Percentage immobility in the forced swim test
No HU	1.5	Plasma	Palmitoleic acid	4	Percentage of time spent in the center of the open field in the object recognition test trial; distance moved in the open field in the object recognition acquisition trial; distance moved in the object recognition test trial	Percentage immobility in the forced swim test
No HU	1.5	Plasma	Sphinganine	5	Percentage of time spent in the center of the open field on the second day of testing; distance moved in the open field on the second day of testing; total time spent exploring the objects in the object recognition training trial; distance moved in the object recognition acquisition trial; distance moved in the object recognition test trial	
HU	0	Cortex	Phospho(enol)pyruvic acid	4	Discrimination index for the object recognition test trial; percentage of time spent in the center of the open field in the object recognition training trial; percentage of time spent in the center of the open field in the object recognition test trial; percentage of time spent in the center of the open field on the second day of testing	
HU	0	Cortex	Spermine	5	Percentage of time spent in the center of the open field on the second day of testing; distance moved in the open field on the second day of testing; percentage of time spent in the center of the open field in the object recognition training trial	Percentage of time spent in the center of the open field on the first day of testing; percentage of time spent exploring the familiar object in the object recognition test trial
HU	0	Plasma	Betaine	4	Percentage of time spent in the center of the open field on the first day of testing; percentage of time spent exploring the novel object in the object recognition test trial	Distance moved in the open field on the first day of testing; distance moved in the open field in object recognition training trial
HU	0	Plasma	Gluconic acid	4	Percentage of time spent exploring the familiar object in the object recognition test trial	Percentage of time spent in the center of the open field on the second day of testing; discrimination index for the object recognition test trial; percentage of time spent in the center of the open field in the object recognition test trial
HU	0	Plasma	N-Acetyl-L-methionine	4	Distance moved in the open field during the first day of testing; distance moved in the object recognition acquisition trial	Percentage of time spent in the center of the open field on the first day of testing; discrimination index for the object recognition test trial
HU	1.5	Cortex	Guanosine 5′-diphosphate	4		Percentage of time spent in the center of the open field on the second day of testing; percentage of time spent in the center of the open field in the object recognition acquisition trial; percentage of time spent in the center of the open field in the object recognition training trial; percentage of time spent in the center of the open field in the object recognition test trial
HU	1.5	Cortex	Maleic acid	4		Percentage of time spent in the center of the open field on the second day of testing; total time spent exploring the objects in the object recognition acquisition trial; percentage of time spent in the center of the open field in the object recognition test trial; percentage of time exploring the familiar object in the object recognition test trial
HU	1.5	Plasma	Alanine	4		Distance moved in the open field on the first day of testing; distance moved in the open field on the second day of testing; distance moved in the object recognition acquisition trial; distance moved in the object recognition test trial
HU	1.5	Plasma	Gluconic acid	5	Percentage immobility in the forced swim test	Distance moved in the open field on the first day of testing; distance moved in the open field on the second day of testing; distance moved in the object recognition acquisition trial; distance moved in the object recognition training trial
HU	1.5	Plasma	Hippurate	4		Distance moved in the open field on the first day of testing; distance moved in the object recognition acquisition trial; distance moved in the open field in the object recognition training trial; distance moved in the open field in the object recognition test trial
HU	1.5	Plasma	Proline	4		Distance moved in the open field on the second day of testing; distance moved in the object recognition acquisition trial; distance moved in the open field in the object recognition training trial; distance moved in the open field in the object recognition test trial
HU	1.5	Plasma	Trigonelline	4		Distance moved in the open field on the first day of testing; distance moved in the open field in the object recognition acquisition trial; distance moved in the open field in the object recognition training trial; percentage of time spent in the center of the open field in the object recognition test trial

^a^ Metabolites that are purely positively associated with behavioral measures are indicated in green; metabolites that are purely negatively associated with behavioral measures are indicated in red; metabolites that are both positively and negatively associated with behavioral measures are indicated in blue.

^b^ N indicates the number of significant associations.

In the sham-irradiated animals without HU, three metabolites in the hippocampus were correlated with the behavioral measures (one purely negatively and two both positively and negatively).

In addition, two metabolites were correlated purely positively with the behavioral measures.

In the animals without HU exposed to 1.5 Gy of radiation, 11 metabolites in the cortex, one metabolite in the hippocampus, and 4 metabolites in the plasma were purely positively correlated with the behavioral measures, while 7 metabolites showed both positive and negative associations.

In the sham-irradiated animals under HU, one metabolite in the cortex was purely positively associated with the behavioral measures, whereas one metabolite in the hippocampus and 3 metabolites in the plasma were both positively and negatively associated with the behavioral measures.

In the animals under HU exposed to 1.5 Gy of radiation, two metabolites in the cortex and four metabolites in the plasma were purely negatively associated with the behavioral measures, while one metabolite in the plasma showed both positive and negative associations.

We next assessed whether any of the 21 metabolites identified in the present study were also among the 40 metabolites in the plasma, hippocampus, or cortex of the simGCRsim-irradiated WAG/Rij rats in our previous study. Phenylalanine and tryptophan were the two metabolites that showed both positive and negative associations with the behavioral measures of both studies (Table 6). Although these metabolites were found in the hippocampus in the previous study on WAG/Rij rats, they were found in the cortex in the present study on Fischer rats. Carnitine in the plasma in the current study showed both positive and negative associations with the behavioral measures in the WAG/Rij rats, while lauroylcarnitine in the cortex and deoxycarnitine in the plasma showed purely positive associations with the behavioral measures in the Fischer rats.

**TABLE 6:** Metabolites shown to be correlated with behavioral measures in our previous simGCRsim-irradiated WAG/Rij animals^a^ that were also found to be associated with the behavioral measures in the present study.

Condition	Dose (Gy)	Compartment	Metabolite^b^	*N* ^c^	Significant positive associations	Significant negative associations
No HU	0	Hippocampus	Phenylalanine	4	Total time spent exploring the objects in the test trial; distance moved in the object recognition test trial; distance moved in the open field on the second day of testing	Percentage immobility in the forced swim test
No HU	0	Hippocampus	Tryptophan	4	Total time spent exploring the objects in the test trial; distance moved in the object recognition test trial; distance moved in the open field on the second day of testing	Percentage immobility in the forced swim test
No HU	1.5	Plasma	Carnitine	4	Total time spent exploring the objects in the test trial; percentage of time spent exploring the familiar object in the object recognition test trial	Total time spent exploring the objects in the object recognition acquisition trial; percentage of time spent in the center of the open field in the object recognition acquisition trial

^a^ The experimental design of the WAG/Rij rat study was slightly different from that of the current study on Fischer rats. In the WAG/Rij study, only a single radiation dose (1.5 Gy) was used while the current study involved two radiation doses (0.75 and 1.5 Gy). In the WAG/Rij rat study, three trials were performed with objects in the object recognition test, namely, an acquisition trial, a training trial, and a test trial; however, the current study included only the training and test trials. In the WAG/Rij rat study, depressive-like behaviors were assessed through the forced swim test, which was not included in the current study.

^b^ Metabolites that were both positively and negatively associated with behavioral measures are indicated in blue.

^c^ N indicates the number of significant associations.

## 4 Discussion

In this study, we characterized the behavioral and cognitive performances of male Fischer rats after sham or total body irradiation with simGCRsim in the absence and presence of HU or control HU. The HU condition is a surrogate measure for weightlessness that is used in simulation studies on Earth (Ray et al., 2001; Morey-Holton and Globus, 2002; Ferreira et al., 2011; Trinel et al., 2013; Kok et al., 2019). The 5-ion, 6-beam simGCRsim radiation source was designed by NASA to provide a simplified radiation field that represents the type of exposure that astronauts are expected to receive from galactic cosmic rays on a Mars mission (Simonsen et al., 2020). In this study, specific behavioral outcomes were measured in response to these two types of spaceflight stressors. In the open field with both absence and presence of objects, there was a radiation × HU interaction in which the effects of radiation were modulated by the HU condition. We evaluated these effects in different body compartments, namely the plasma, hippocampus, and cerebral cortex.

The effects of radiation analyzed in the plasma of animals without HU or control HU showed that the riboflavin metabolic pathway was the most affected, while the glycerophospholipid, sphingolipid, and glutathione metabolic pathways were less affected when comparing sham irradiation vs. 0.75 Gy exposure. The dose of 0.75 Gy is directly relevant to the exposure conditions predicted for a 3-year Mars mission for a human (Hassler et al., 2014; Simonsen et al., 2020). Comparing sham irradiation vs. a higher anchor dose of 1.5 Gy of simGCRsim exposure in animals without HU or control HU, the riboflavin metabolic pathway was found to be affected, confirming the results at the lower, space-relevant dose. In addition, arginine biosynthesis, glutamine and glutamate metabolism, as well as alanine, aspartate, and glutamate metabolic pathways were affected. Riboflavin, glutamine, and arginine levels were higher in the plasma of animals irradiated with 1.5 Gy than the sham-irradiated animals. When the effects of radiation were analyzed in the plasma in animals under the control HU condition, the riboflavin metabolic pathway was found to be the most strongly affected, with the glutathione metabolic pathway being less affected. The effects of the control HU condition on the plasma of sham-irradiated animals were analyzed through comparisons with animals without HU, and the alanine, aspartate, and glutamate metabolism, riboflavin metabolism, arginine biosynthesis, as well as glutamine and glutamate metabolism were affected.

The metabolic outcomes in different regions of the brain were also considered directly to evaluate links to behavioral patterns. When the effects of 0.75 Gy irradiation exposure were analyzed in the hippocampus of animals under the control HU condition, the glutamine and glutamate metabolic pathway was found to be affected. In the sham-irradiated animals, the effects of the control HU condition on the hippocampus were analyzed through comparisons with those without HU, which showed that the phenylalanine, tyrosine, and tryptophan pathway was the most affected, followed by the riboflavin metabolic pathway. When the effects of 0.75 Gy irradiation exposure were analyzed in the cerebral cortex of animals under the control HU condition, the glutamine and glutamate metabolic pathway was affected similar to that in the hippocampus, while animals without the control HU condition showed changes in the riboflavin pathway. For the sham-irradiated animals, the effects of the control HU condition on the cortex were analyzed through comparisons with those without HU, which revealed that the riboflavin metabolic pathway was affected. In the 0.75-Gy-irradiated animals, the glutamine and glutamate metabolic pathway was affected, while the riboflavin metabolic pathway was affected in the 1.5-Gy-irradiated animals. The distinctly affected pathways between the control HU 0.75-Gy- vs. 1.5-Gy-irradiated animals may be related to their differential effects on the behavioral performances in the open field in the absence and presence of objects.

The levels of 4/5-oxo-proline were higher in animals with HU than those without HU; 4-hydroxyproline (which when oxidized becomes oxo-proline) is formed by prolylhydroxylase (with vitamin C as the cofactor) in procollagen tissues. If the mature, crosslinked collagen breaks down, then 4-hydroxyproline is released. The higher levels of 4/5-oxo-proline in HU animals may reflect the collagen breakdown in the hippocampus linked to neuronal injury (Wareham et al., 2024). Although we noted both 4- and 5-oxo-proline in our library, we cannot distinguish between them and have therefore denoted it as 4/5-oxo-proline.

A total of 21 plasma metabolites were correlated with the behavioral measures, of which twelve associations were purely positive and one association was purely negative; furthermore, eight metabolites were both positively and negatively associated with the behavioral measures. The integrated analysis indicates that the plasma and brain biomarkers associated with behavioral and cognitive performance are dependent on the environmental conditions experienced (i.e., HU, control HU or simulated space radiation).

We were especially interested in the interactions between simGCRsim exposure and each of the HU conditions. Our new data show that the groupings of both the phenylalanine, tyrosine, and tryptophan metabolism as well as phenylalanine metabolism and biosynthesis exhibit very strong pathway changes after simGCRsim irradiation and in the control HU condition. Remarkably, the phenylalanine, tyrosine, and tryptophan metabolic pathway was also the most affected by simulated space radiation and HU in the plasma, hippocampus, and cortex of WAG/Rij rats (Raber et al., 2021) and by photon irradiation in Fischer rats (Raber et al., 2024). In addition, the phenylalanine and tryptophan levels were associated with better behavioral measures in both WAG/Rij and Fischer rats. Higher phenylalanine and tryptophan levels in the cortexes of Fischer rats without HU exposed to 0.75 Gy of radiation were associated with reduced measures of anxiety in the open field. Higher phenylalanine and tryptophan levels in the hippocampi of sham-irradiated WAG/Rij rats without HU were associated with reduced depressive-like behaviors in the forced swim test. Tryptophan and tyrosine play executive functional roles (Aquili, 2020) and were earlier shown to be negatively affected by X-ray irradiation (Davis et al., 2014; Hienz et al., 2008; Tang et al., 2022). Tryptophan is the precursor of serotonin, and elevated dietary tryptophan levels were shown to suppress post-stress plasma glucocorticoid levels in another study (Hoglund et al., 2019).

HU had detrimental effects on activity and freezing levels in the open field as well as on the measures of anxiety in the elevated plus maze. These findings are consistent with the reported detrimental effects of simulated microgravity on three-dimensional visuospatial tuning and orientation in mice (Oman, 2007). Additionally, phenylalanine and tyrosine (found to be elevated by HU) are precursors of norepinephrine (Fernstrom and Fernstrom, 2007); elevated norepinephrine levels are indicators of stress (Koob, 1999) and may have contributed to the anxiety-like behaviors observed in HU rats. In the present study, sham-irradiated mature male Fischer rats under HU showed spatial habituation learning in the open field and moved less on day 2 than day 1. In contrast, sham-irradiated male WAG/Rij rats of a similar age under HU showed impaired spatial habituation learning (Raber et al., 2021). These data suggest that Fischer rats may be less susceptible to HU than WAG/Rij rats.

Two metabolites were found to be associated with the behavioral measures between the current simGCRsim and earlier photon studies with mature male Fischer rats (Raber et al., 2024), namely succinate and 5-methylcytosine. Succinate levels in the hippocampus in the present study and in the plasma in the previous study (Table 4) were positively correlated with the activity levels and reduced anxiety-like behaviors (time spent in the center of the open field without and with objects). Owing to COVID-19-related travel restrictions when vaccines were not available, we were unable to travel to the Medical College of Wisconsin to euthanize and dissect the brains of the rats. We recognize that correlations with the behavioral measures involved the same metabolites in distinct compartments between the two studies; consistent with these results, succinate feeding enhanced the endurance exercise capacities of mice (Xu et al., 2022) while succinic acid administration had anxiolytic effects in the mice (Gang et al., 2009). The second metabolite was 5-methylcytosine, whose presence in the plasma suggests that there was modification and degradation of the DNA. Consistent with this notion, the positive correlation between plasma 5-methylcytosine and increased activity levels as well as reduced measures of anxiety were noted in the control HU mice exposed to 0.75 Gy of radiation in the present study, along with increased activity levels and cognitive performance in the object recognition test in rats exposed to 8 Gy of photons in the absence of HU in the previous study (Table 4). These results are consistent with the simulated space irradiation-induced alterations in hippocampal DNA methylation reported in mice (Impey et al., 2016a, 2016b, 2017, 2023; Torres et al., 2019). These results indicate that 5-methylcytosine may specifically be a good plasma biomarker of behavioral and cognitive performances following irradiation.

Lauroylcarnitine is important for energy metabolism to convert fat into energy, and increased lauroylcarnitine levels have been shown to slow disease progression in various cognitive and behavioral conditions like schizophrenia (Zhao et al., 2023), Alzheimer’s disease (Kepka et al., 2020), autism spectrum disorders (Kepka et al., 2021), traumatic brain injury (Sharma et al., 2024), and liver cirrhosis (Li and Zhao, 2021). Consistent with these patterns, there were positive correlations between the cortical levels of lauroylcarnitine and behavioral performance in the sham-irradiated animals without HU for the object recognition test. Lauroylcarnitine is also known to improve exercise performance (Mielgo-Ayuso et al., 2021; Vecchio et al., 2021); accordingly, plasma lauroylcarnitine levels were positively correlated with enhanced activity and reduced anxiety levels in the control HU animals exposed to 0.75 Gy of radiation.

The level of sphingosine-1-phosphate generated by degradation of ceramides is higher in the brain than any other organ and lower in the cortex than the spinal cord, brainstem, or cerebellum (Jiang and Han, 2006). Sphingosine-1-phosphate has been reported to have both neuroprotective and neurotoxic effects that are hypothesized to depend on the amount and subcellular site of generation. For example, sphingosine-1-phosphate modulates calcium homeostasis, synaptic transmission, epigenetic regulation, and integrity of the blood–brain barrier such that reduced levels are noted in schizophrenia, while the release of sphingosine-1-phosphate from astrocytes has been reported to impair autophagy (Xiao et al., 2023), be proinflammatory [please see van Echten-Deckert (2023) for a review], and play a role in the development and progress of Parkinson’s disease (Wang et al., 2023). Consistent with the role of sphingosine-1-phosphate in synaptic transmission, its cortical levels were positively correlated with the behavioral measures in the object recognition test in the sham-irradiated animals without HU.

In an earlier work, exogenous administration of sodium benzoate was shown to increase the measures of anxiety and impair the activity levels, exploratory behaviors, and memory in rats (Asejeje et al., 2022). In contradistinction to the findings in rats, sodium benzoate has been shown to improve cognition in women with dementia (Lin et al., 2021). In the current study, hippocampal levels of sodium benzoate in rats exposed to 1.5 Gy of radiation without HU were positively correlated with activity and reduced anxiety levels in the open field without and with objects. These results suggest that following an environmental challenge like simulated space irradiation or a severe neurological condition, increased levels of sodium benzoate may be beneficial for behavioral performance. However, it is also conceivable that the beneficial effects of sodium benzoate are evident only at lower doses than those typically used in pharmacological studies.

A combination of metabolic activators (nicotinamide riboside, N-acetyl-L-cysteine, and L-carnitine tartrate) has been shown to improve cognition in patients with Alzheimer’s disease; this cognitive improvement was also associated with increased plasma levels of deoxycarnitine (Yulug et al., 2023). Consistent with this finding, the plasma deoxycarnitine levels in male Fischer rats exposed to 1.5 Gy of radiation without HU in this study were positively correlated with activity levels in the open field and cognitive performance in the object recognition test.

In earlier work by other researchers, the level of sphingomyelin 18:0 in the hippocampus was associated with better cognitive performance in the Y maze and object recognition test (Kalinichenko et al., 2022). Consistent with this finding, the plasma sphingomyelin levels in animals exposed to 1.5 Gy of radiation without HU in the present study were positively correlated with activity levels and cognitive performance in the object recognition test.

Supplementation with citrulline, a byproduct of NOS-mediated conversion of arginine to nitric oxide, has been shown to enhance athletic performance (Gentilin et al., 2022), prevent age-related decline in long-term potentiation (Ginguay et al., 2019), and improve cognitive performance in animal models of neurological conditions (Martinez-Gonzalez et al., 2021; Yabuki et al., 2013). Consistent with these results, the plasma citrulline levels were positively correlated with increased activity and reduced anxiety levels in the control HU animals exposed to 0.75 Gy of radiation. Citrulline appears to reflect NO synthesis, which increases during activity and may be related to greater blood flow in muscles.

Lauroylcarnitine has been shown to improve exercise performance (Mielgo-Ayuso et al., 2021; Vecchio et al., 2021). Consistent with this result, the plasma lauroylcarnitine levels that are potentially markers of beta oxidation of fatty acids were positively correlated with enhanced activity and reduced anxiety levels in the control HU animals exposed to 0.75 Gy of radiation.

In contrast to the positive associations of plasma deoxycarnitine and cortical lauroylcarnitine levels with the behavioral measures in Fischer rats in the present study, higher levels of carnitine in the plasma of WAG/Rij rats exposed to 1.5 Gy of radiation without HU were positively associated with the total time spent exploring objects and percentage of time exploring the familiar object in the object recognition test trial but negatively associated with the total time spent exploring the objects in the object recognition acquisition trial. Deoxycarnitine is a direct precursor of carnitine and is involved with tissue exchange with carnitine (Sartorelli et al., 1989). These data suggest that the beneficial effects of the carnitine pathway may be dependent on the genetic background and/or dose-dependent.

Serum levels of pipecolic acid were found to be decreased and anxiety levels increased in patients with active ulcerative colitis (Yuan et al., 2021). Consistent with this result, the plasma pipecolic acid levels were positively correlated with enhanced activity and reduced anxiety levels in the control HU animals exposed to 0.75 Gy of radiation.

In the present study, the plasma and brain tissue samples were analyzed 9 months after sham irradiation or total body irradiation in the absence or presence of HU and 7 months after the behavioral and cognitive performance evaluations. Together with our earlier study on mature male WAG/Rij rats following simulated space radiation in the absence and presence of HU, the metabolic pathways affected by HU and to a lesser extent by photon irradiation as well as the relationships between the behavioral measures and individual metabolite levels in plasma support the feasibility of developing stable long-term biomarkers of the responses to HU as well as behavioral and cognitive performance.

In summary, the detrimental effects of HU on behavioral and cognitive performance as well as metabolic pathways in plasma illustrate the importance of developing mitigators to reduce the effects of microgravity and space radiation on the brain functions of astronauts during and after space missions. The integrated analysis in this study indicates that plasma and brain biomarkers associated with behavioral and cognitive performance are dependent on the environmental conditions experienced (i.e., HU, control HU, or simulated space radiation). The metabolomics data of the current study on Fischer rats and previous study on WAG/Rij rats suggest that it may be possible to develop stable plasma biomarkers of HU, simulated space radiation, as well as behavioral and cognitive performances that could be used to develop and test such mitigators. Phenylalanine, tyrosine, and tryptophan metabolism as well as the metabolites phenylalanine and tryptophan in plasma are especially good candidates for mitigation to consider for spaceflights as these were found in both the Fischer and WAG/Rij rats exposed to simGCRsim and/or HU.

## Data Availability

The original contributions presented in the study are included in the article/Supplementary Material; further inquiries can be directed to the corresponding author.
