# Supplementary material for: Effects of 5-ion 6-beam sequential irradiation in the presence and absence of hindlimb or control hindlimb unloading on behavioral performances and plasma metabolic pathways of Fischer 344 rats
Source: Front Physiol. 2024 Nov 13;15:1486767. doi: 10.3389/fphys.2024.1486767 (PMC11598337; doi:10.3389/fphys.2024.1486767)
Supplement: Supplementary file 2 [file Table1.docx]

Supplementary Material

**
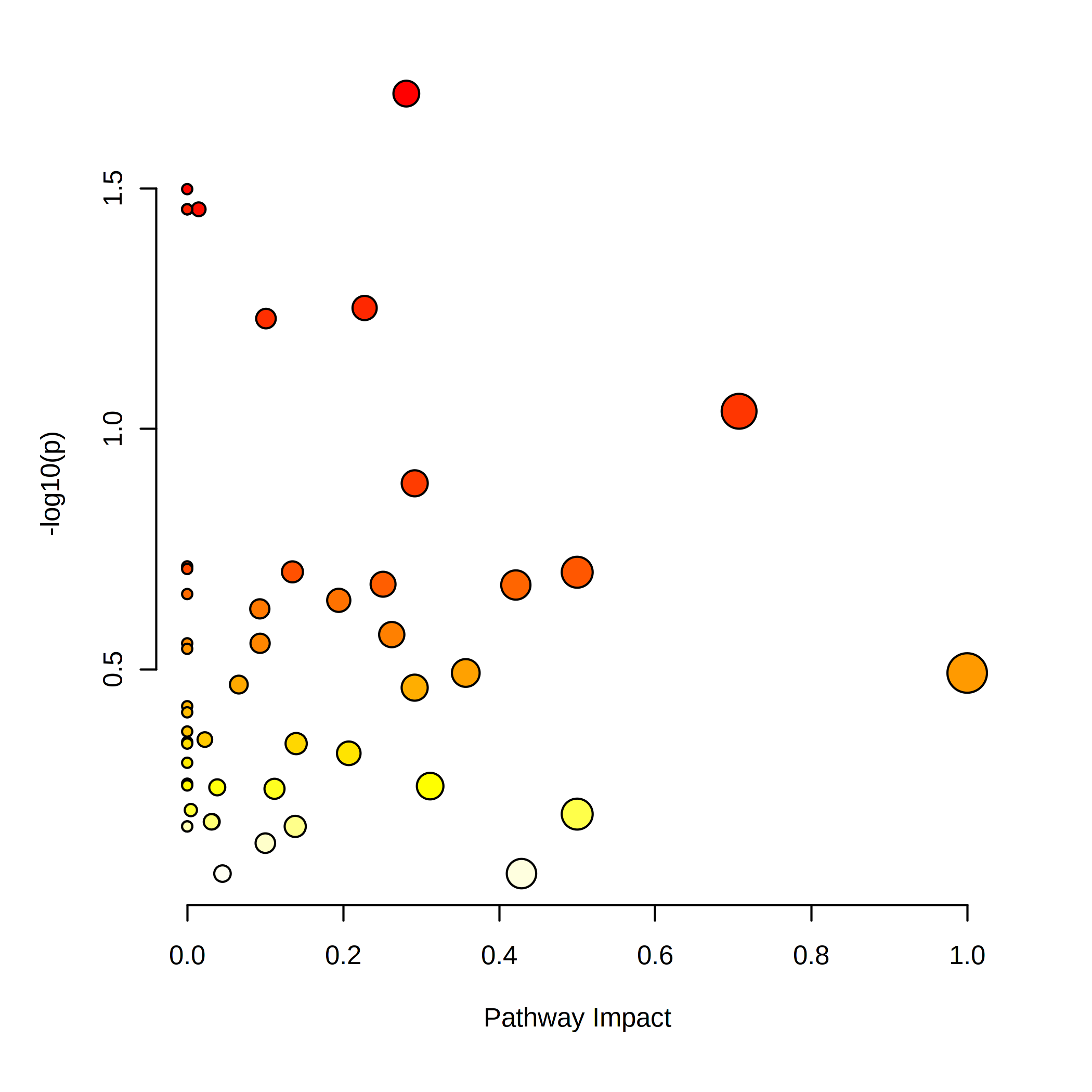
**

**Purine metabolism**

**Ether lipid metabolism**

**Fatty acid elongation/Fatty acid degradation/Biosynthesis of unsaturated fatty acids**

**Pyrimidine metabolism**

**Glycerophospholipid metabolism**

**Fatty acid biosynthesis**

**Supplementary Figur**

| **Pathway Name** | ***p*** | **Impact** |
| --- | --- | --- |
| **Purine metabolism** | **0.020074** | **0.28092** |
| **Fatty acid biosynthesis** | **0.034936** | **0.01472** |
| **Pyrimidine metabolism** | **0.056045** | **0.22746** |
| **Glycerophospholipid metabolism** | **0.058963** | **0.10099** |

**Supplementary Figure 1.** When effects of radiation were analyzed in the hippocampus of animals that did not receive HU or the HU control condition, no pathways were affected in animals irradiated with 0.75 Gy and no pathway with an impact of 0.5 or higher in animals irradiated with 1.5 Gy. The Purine metabolism and Pyrimidine pathways were affected with an impact of 0.28 and 0.23, respectively.

| **Pathway Name​** | **p​** | **FDR​** |
| --- | --- | --- |
| **Fatty acid biosynthesis​** | **0.057551​** | **0.58821​** |

**
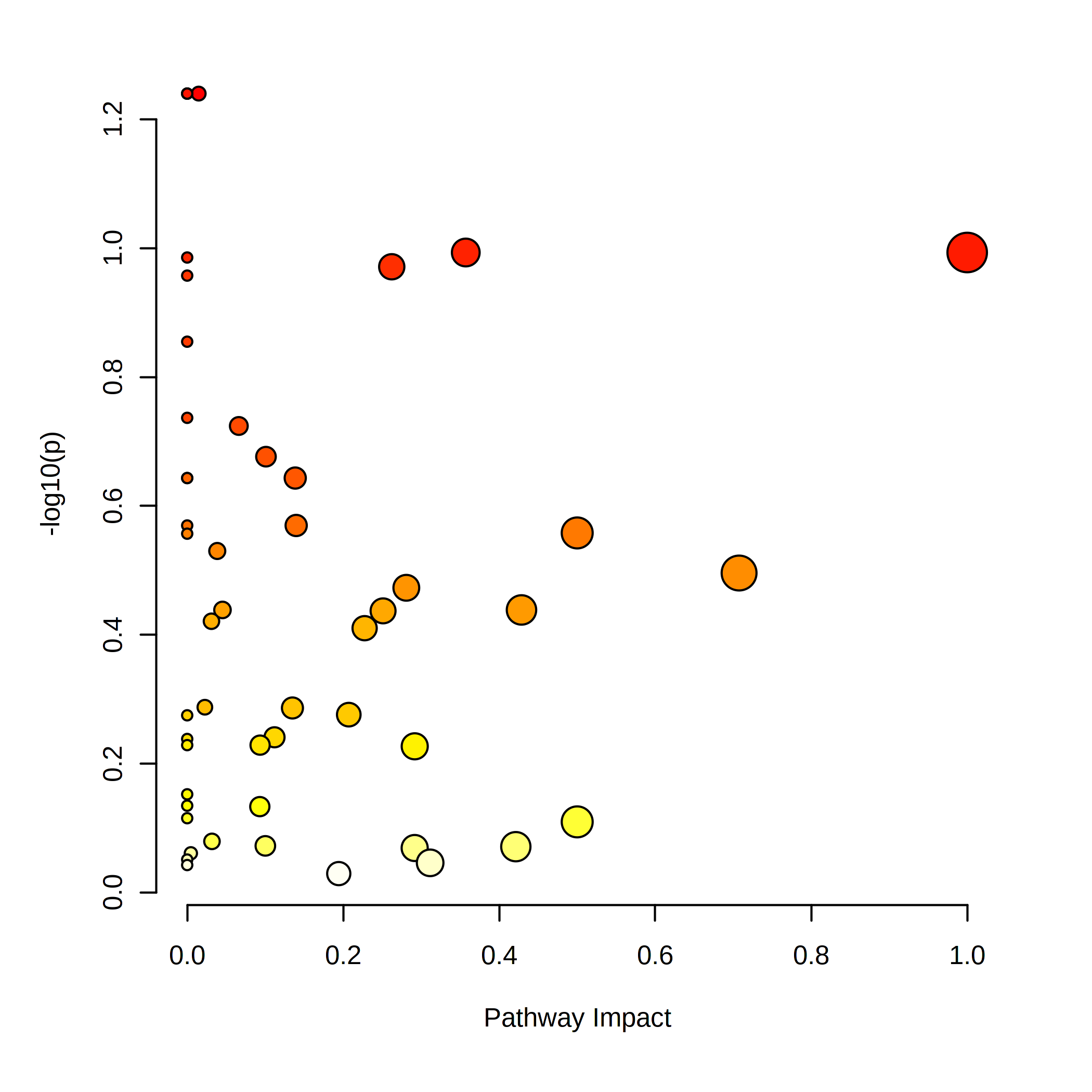
**

**Fatty acid biosynthesis**

**Fatty acid elongation/fatty acid degradation/Biosynthesis of unsaturated fatty acids**

**Supplementary Fig. 2.** When effects of 1.5 Gy irradiation in the hippocampus of animals that did receive the HU control condition, no pathways were affected.

**A**

| **Pathway Name** | **p** | **FDR** |
| --- | --- | --- |
| **Purine metabolism** | **0.014814** | **0.40412** |
| **Pantothenate and CoA biosynthesis** | **0.01777** | **0.40412** |
| **Propanoate metabolism** | **0.030461** | **0.40412** |
| **Sphingolipid metabolism** | **0.07524** | **0.40412** |

**B**

**Supplementary Fig. 3.** When effects of radiation were analyzed in the cortex of animals that did not receive HU or the HU control condition, no pathways with an impact of 0.5 or greater were affected in animals irradiated with 0.75 (**A**) or 1.5 Gy (**B**).

| **Pathway Name​** | ***p​*** | **FDR​** | **Impact​** |
| --- | --- | --- | --- |
| **Fatty acid biosynthesis​** | **0.057551​** | **0.58821​** | **0.01472​** |

**Supplementary Figure 2.** When effects of 1.5 Gy irradiation in the hippocampus of animals that did receive the HU control condition, no pathways were affected.

**A**


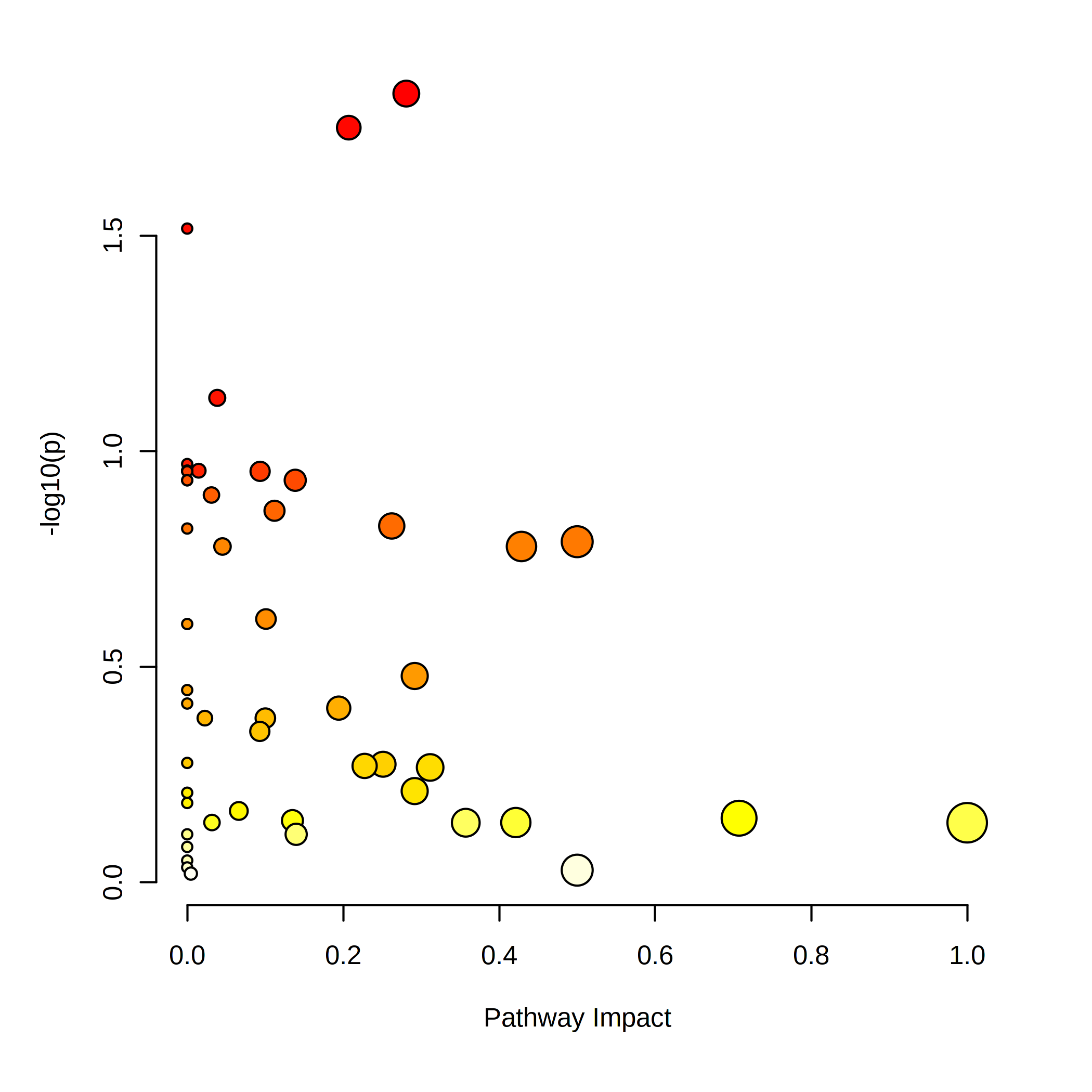


Purine metabolism

Pantothenate and CoA biosynthesis

Propanoate metabolism

Sphingolipid metabolism:

| **Pathway Name** | ***p*** | **FDR** | **Impact** |
| --- | --- | --- | --- |
| **Purine metabolism** | **0.014814** | **0.40412** | **0.28092** |
| **Pantothenate and CoA biosynthesis** | **0.01777** | **0.40412** | **0.20714** |
| **Propanoate metabolism** | **0.030461** | **0.40412** | **0.0** |
| **Sphingolipid metabolism** | **0.07524** | **0.40412** | **0.03854** |

**B**


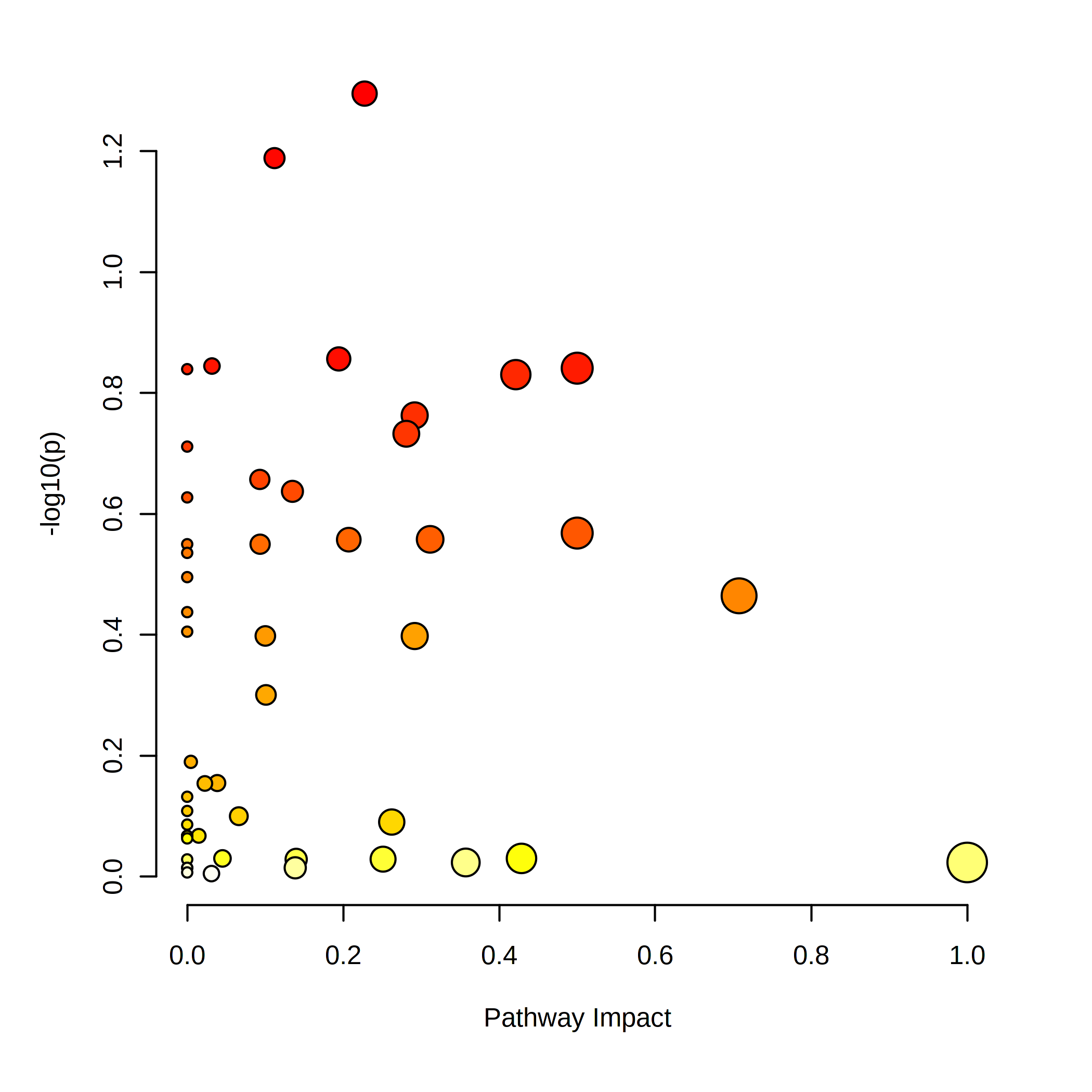


Pyrimidine metabolism

beta-Alanine metabolism

| **Pathway Name** | ***p*** | **FDR** | **Impact** |
| --- | --- | --- | --- |
| **Pyrimidine metabolism** | **0.050661** | **0.78229** | **0.22746** |
| **beta-Alanine metabolism** | **0.064773** | **0.78229** | **0.11194** |

**Supplementary Figure 3.** When effects of radiation were analyzed in the cortex of animals that did not receive HU or the HU control condition, no pathways with an impact of 0.5 or greater were affected in animals irradiated with 0.75 (**A**) or 1.5 Gy (**B**).
